# Protein–protein interaction network module changes associated with the vertebrate fin-to-limb transition

**DOI:** 10.1038/s41598-023-50050-2

**Published:** 2023-12-18

**Authors:** Pasan C. Fernando, Paula M. Mabee, Erliang Zeng

**Affiliations:** 1 Department of Plant Sciences, University of Colombo, Colombo, Sri Lanka; 2 Department of Biology, University of South Dakota, Vermillion, SD USA; 3 Departments of Preventive & Community Dentistry, College of Dentistry, University of Iowa, Iowa City, IA USA; 4 Division of Biostatistics and Computational Biology, College of Dentistry, University of Iowa, Iowa City, IA USA; 5 Departments of Biostatistics, College of Public Health, University of Iowa, Iowa City, IA USA; 6 Departments of Biomedical Engineering, College of Engineering, University of Iowa, Iowa City, IA USA; 7 National Ecological Observatory Network, Battelle, 1625 38th St. #100, Boulder, CO 80301 USA

**Keywords:** Network topology, Proteome informatics

## Abstract

Evolutionary phenotypic transitions, such as the fin-to-limb transition in vertebrates, result from modifications in related proteins and their interactions, often in response to changing environment. Identifying these alterations in protein networks is crucial for a more comprehensive understanding of these transitions. However, previous research has not attempted to compare protein–protein interaction (PPI) networks associated with evolutionary transitions, and most experimental studies concentrate on a limited set of proteins. Therefore, the goal of this work was to develop a network-based platform for investigating the fin-to-limb transition using PPI networks. Quality-enhanced protein networks, constructed by integrating PPI networks with anatomy ontology data, were leveraged to compare protein modules for paired fins (pectoral fin and pelvic fin) of fishes (zebrafish) to those of the paired limbs (forelimb and hindlimb) of mammals (mouse). This also included prediction of novel protein candidates and their validation by enrichment and homology analyses. Hub proteins such as *shh* and *bmp4*, which are crucial for module stability, were identified, and their changing roles throughout the transition were examined. Proteins with preserved roles during the fin-to-limb transition were more likely to be hub proteins. This study also addressed hypotheses regarding the role of non-preserved proteins associated with the transition.

## Introduction

Phenotypes, including fin development and limb development, emerge from the intricate interplay of numerous genes and proteins within complex biological pathways^1–3^. Evolutionary shifts in phenotypes, spurred by environmental or other changes, entail modifications in protein interactions and their pathway associations. Most often, it is the intricate network of protein interactions, rather than the contribution of a single protein, that determines the resulting phenotype^1,4^. Thus, the assembly of proteins and their interactions, i.e., modular protein structure^1^, is vital for understanding the evolutionary mechanisms that drive phenotypic changes in the field of evolutionary biology. The analysis of protein modules has become common in bioinformatics, and the concept of modular evolution has emerged to explain the changes occurring in groups, rather than individual proteins, during the evolution of organisms^5–7^. However, most studies have concentrated on smaller protein complexes, typically containing less than 20 proteins, which determine molecular functions^8–10^. In contrast, phenotypes such as fins and limbs, are shaped by a multitude of proteins with diverse molecular functions belonging to various biological pathways. The majority of previous protein network studies centered on phenotypes have targeted human diseases^1,11^. To our knowledge, there have been no studies of modules aimed at understanding evolutionary transitions in phenotypes. Given the profound anatomical changes associated with vertebrate evolution, such as the transformation from fins to limbs, understanding the bases for both the conservation and the changes are critical. This study uses the fin to limb transition in vertebrate evolution as a case study for the value of employing PPI networks in better understanding the molecular basis of evolutionary changes in phenotypes.

The fin-to-limb transition is an iconic anatomical change associated with the evolution of terrestrial vertebrates from aquatic fish-like ancestors^12,13^. According to fossil record, the transformation of fishes into land vertebrates began in the Devonian, 365–408 million years ago^13^. This well-studied transformation is associated with numerous phenotypic changes beyond the shift from fin to limb^14^, such as modifications in the cranial and axial skeleton^15^. Homologies between the anatomical structures of land and aquatic vertebrates are evident from numerous shared characteristics. For instance, the pectoral fin endoskeleton of panderichthyid fish fossils shows substantial similarities to the limb skeletons of terrestrial tetrapods, such as the presence of a proximal humerus and two distal bones^12^. This and other evidence support the concept that forelimbs and hindlimbs of tetrapods are homologous to the pectoral and pelvic fins of fishes, respectively.

Identifying the genetic changes associated with the fin-to-limb transition is a frequent subject of comparative study in evolutionary and developmental biology, with a long and continuing legacy of experimental endeavors^16–21^. While many wet lab experiments have demonstrated the evolutionary importance of key genes such as *shh*^12,16^, computational studies focusing on the fin-to-limb transition have been relatively few^17^. The emergence of large protein–protein interaction (PPI) networks offers a productive way forward to understand the specific changes in biological networks associated with phenotypes altered during such evolutionary transitions. As major anatomical changes involve many proteins and pathways, the application of PPI networks enables a systems biology perspective.

This research sought to isolate functional modules^6,22^, i.e., a set of nodes that are highly connected internally and sparsely connected with external nodes^1^ that correspond to fins and limbs. A variety of functional module detection algorithms exist^5^. For modules associated with complex phenotypes, which typically involve a large number of proteins, it can be advantageous to perform module detection using prior knowledge as a computational constraint^1,4,23^. These methods begin with a set of proteins known to be associated with a given phenotype and expand the module based on the network structure. One of the simplest ways for isolating a functional module by expansion involves incorporating all immediate neighbors of the proteins associated with the known phenotype into the module^1^. However, this method has been found to yield a high number of false positives^1^. As a result, more accurate network-based candidate protein prediction algorithms, such as the Hishigaki method^24^, are often used to predict new candidate proteins for module inclusion^1,4,24^. The Hishigaki method mitigates the bias towards extensively studied functions and was used herein.

This study further sought to identify hub proteins in the functional modules, and to compare them between fins and limbs. Hub proteins typically have more interactions than others in the module^3,25,26^ and function to maintain module stability; their removal is likely to disrupt module organization, and subsequently the biological function(s) or phenotype(s) that is regulated. Characterizing how hub proteins change over the course of evolution while maintaining developmental stability is a key question in evolutionary developmental biology. To date, a primary focus has been on identifying the proteins that are conserved throughout evolution and their organization within respective modules^8,10^. It has been hypothesized that evolution navigates gradual modular changes, preserving fundamental structure, because dramatic alterations in protein interactions jeopardize organismal function^7^. Supporting this, conserved proteins are observed to play an important role in maintaining the stability of protein modules during evolution^7,8,10^. The recruitment and the removal of other proteins and the rewiring of biological pathways are often held together by the conserved proteins. Module analysis allows identification of these crucial conserved proteins, which often also serve as hub proteins^7,8^. While these may play a role in maintaining protein module structure, species-specific module proteins recruited or removed during evolution can also have essential roles contributing to evolutionary transitions^27^. This study aimed to distinguish conserved and species-specific proteins in functional modules as a potential mechanism to better understand their roles in evolution.

## Methods

The goal of this work was to compare PPI network modules responsible for paired fins with those of paired limbs to study the modular changes associated with the transition. Zebrafish and mouse were selected as model organisms to extract modules from their PPI network for paired fins and paired limbs, respectively. Ensuring the quality of PPI network data remains a significant challenge in research endeavors involving PPI networks^4,28^. The PPI networks constructed using experimental methods, such as the high-throughput yeast two-hybrid assay, often contain a multitude of spurious interactions^4,28,29^. This necessitated refining the raw PPI networks to bolster the precision of our current network analyses. In addressing this need, we used enhanced integrated networks developed in our previous research^28^. These were formulated by integrating raw PPI data from the STRING database^30^ with established experimental insights on protein-anatomy relationships, sourced from the Monarch Initiative repository^31^, which archives proteins annotated using Uberon anatomy ontology terms^32^. This integration improves the quality of raw PPI networks by filtering out spurious interactions, leveraging the highly accurate protein-anatomy relationships documented through trustworthy experimental knowledge^28^. Our earlier research validated the robustness of these integrated networks, highlighting their superiority over raw PPI networks in predicting new anatomical protein candidates, such as those for fins and limbs^28^. Consequently, we selected the most effective integrated networks for zebrafish and mouse, identified in our previous research^28^, to carry out the module analyses for this study.

**Figure 1:**
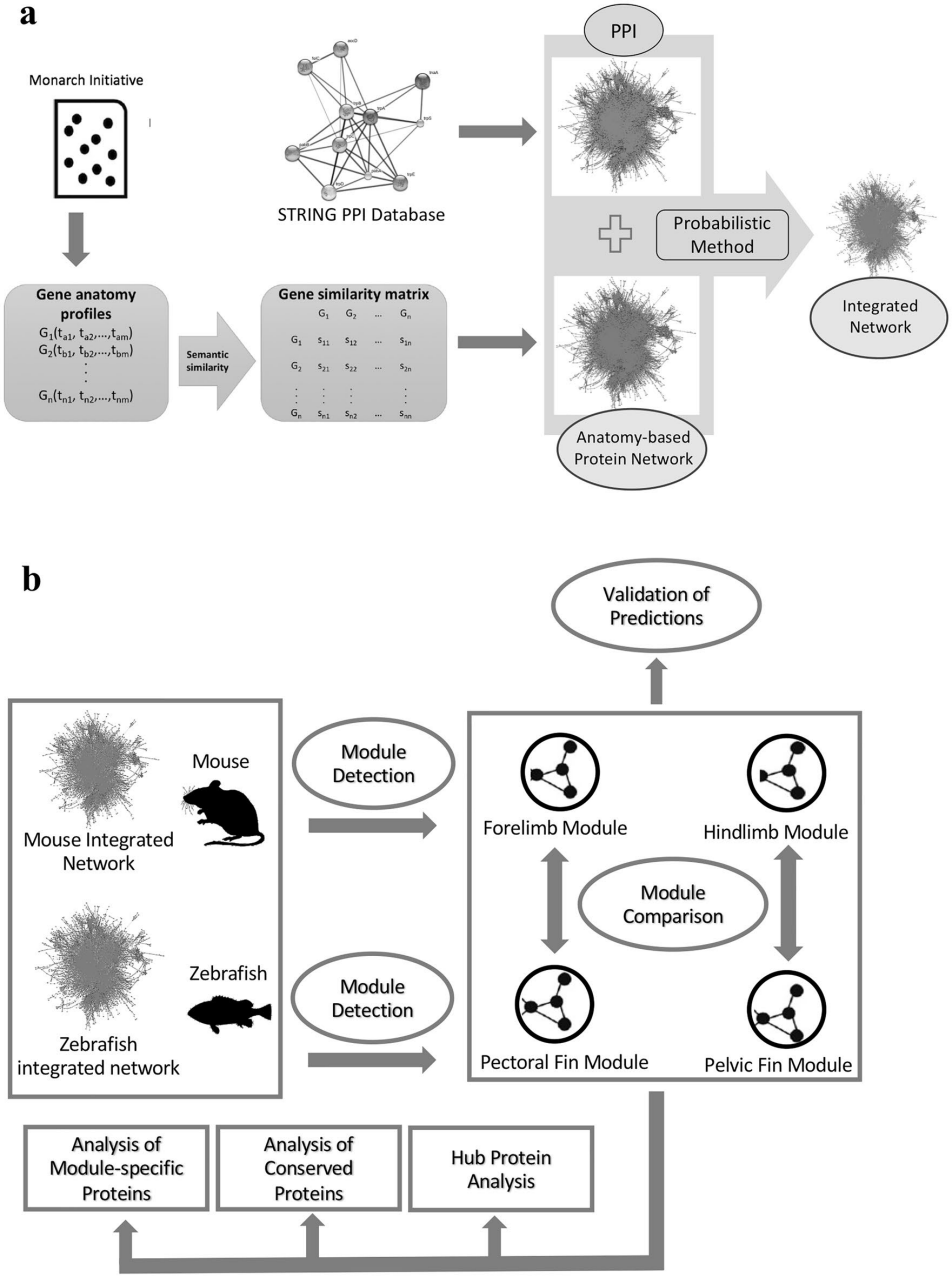
The comprehensive workflow used for comparing paired fin modules with corresponding paired limb modules. Part (**a**) illustrates the construction of integrated networks by integrating PPI networks with anatomy-based protein networks, and part (**b**) represents the use of species-specific integrated networks to detect and compare the paired fin modules of zebrafish with the paired limb modules of mouse.

In this study, we constructed a network workflow for comparing paired fin modules to paired limb modules. The initial phase of this workflow entails the fusion of PPI networks from the STRING database with anatomical annotations sourced from the Monarch Initiative repository to generate integrated networks. This integration serves to enhance network quality, and the most effective networks were then chosen based on our prior findings^28^. Then, the PPI modules pertaining to paired fins and limbs were extracted from integrated zebrafish and mouse networks, respectively, using the Hishigaki network prediction algorithm^24^. This extraction process predicts new protein candidates for each fin and limb module based on originally annotated proteins. Subsequently, the accuracy of these newly projected protein candidates was verified through enrichment and homology analyses. The concluding phase involved comparing the zebrafish paired fin modules with the mouse paired limb modules, facilitating the examination of modular changes. This comparison provided insights into conserved versus module-specific proteins and their shifting significance during the evolutionary transition. These workflow steps are visually represented in Fig. 1 and the Python scripts used are available at: 10.5281/zenodo.4445583. It is noteworthy to mention that while the current workflow targets the paired fin-limb comparison, the provided scripts are adaptable for any anatomical entity defined in the Uberon ontology and for any network sourced from the STRING database.

### Generation and selection of the integrated networks for module detection

During the integrated network generation step (Fig. 1), which was conducted during our previous work^28^, anatomy-based protein networks were constructed by calculating the semantic similarity between anatomy ontology terms annotated to different proteins using four semantic similarity methods (Lin^33^, Resnik^34^, Schlicker^35^, and Wang^36^). Subsequently, these semantic networks were integrated with the PPI networks for zebrafish and mouse. During this process, the combined STRING PPI network score for each interaction was integrated with the equivalent semantic similarity scores of protein pairs from the anatomy-based semantic networks. This network integration substantially increased the candidate protein prediction accuracy for anatomical entities, thus ensuring these integrated networks are superior for module detection than raw PPI networks retrieved from the STRING^22,23,28,37,38^. Furthermore, of the four integrated networks constructed by the four semantic similarity methods, the best-performing semantic networks for zebrafish and mouse were evaluated in our previous work. This assessment involved using the Hishigaki network-based candidate protein prediction method^24,28^ coupled with leave-one-out cross-validation process^28^. The two best-performing networks for zebrafish (Lin) and mouse (Schlicker) were used in this study to ensure the accuracy of module detections. Hereafter, these will be referred as “zebrafish integrated network” and “mouse integrated network”. Both Lin (Eq. 1) and Schlicker (Eq. 2) methods consider the Information Content (IC) of each term of the anatomy ontology, factoring in the number of protein annotations for each term.

1 $$sim_{L } \left( {t_{1} ,t_{2} } \right) = \mathop {\max }\limits_{{t \in S\left( {t_{1} ,t_{2} } \right)}} \left\{ {\frac{2IC\left( t \right)}{{IC\left( {t_{1} } \right) + IC\left( {t_{2} } \right)}}} \right\}$$

2 $$sim_{S } \left( {t_{1} ,t_{2} } \right) = \mathop {\max }\limits_{{t \in S\left( {t_{1} ,t_{2} } \right)}} \left\{ { \frac{2IC\left( t \right)}{{IC\left( {t_{1} } \right) + IC\left( {t_{2} } \right)}} \left( {1 + IC\left( t \right)} \right)} \right\}$$

In the above equations, *t*_*1*_ and *t*_*2*_ represent the ontology terms for which the similarity is being calculated, whereas *S* denotes the set of common ancestors for these two terms. The IC for a specific term *t* is represented by *IC(t)*, and is calculated based on the number of proteins annotated to term *t* as illustrated below (Eqs. 3 and 4).

3 $$IC\left( t \right) = - log\left( {p\left( t \right)} \right)$$

4 $$P\left( t \right) = \frac{{Number\, of\, proteins \,associated \,with \,the \,term\, {\text{t}} + constant}}{Total\, number \,of \,proteins \,associated \,with \,the \,entire \,ontology}$$

### Detection of network modules

For module detection, proteins with direct annotations to the pectoral fin (UBERON:0000151), forelimb (UBERON:0002102), pelvic fin (UBERON:0000152), and hindlimb (UBERON:0002103) were used as prior information, and their anatomical profiles were extracted from the Monarch Initiative repository (https://monarchinitiative.org/; 06/20/2018)^31^. In addition, proteins that were annotated to the parts (e.g., pectoral fin radial skeleton is a part of the pectoral fin) and developmental precursors (e.g., pectoral fin bud and forelimb bud) of the above entities were extracted using the Uberon anatomy ontology^32^ relationships. The proteins directly annotated to the anatomical entity of interest or annotated to a part or a developmental precursor of the entity are collectively referred to as “proteins with original annotations”.

The process of module identification begins with proteins bearing original annotations. The Hishigaki network-based candidate protein prediction method^24,28^ is then used to identify potential additional proteins relevant to the anatomical entities of interest. The Hishigaki method employs a chi-square-based scoring algorithm to estimate the probability of a given protein having a particular function. This approach mitigates the bias towards extensively studied functions, where higher scores are often assigned due to a greater number of original annotations. Consequently, the Hishigaki method serves as an ideal candidate for extracting protein modules for anatomical entities, such as fin and limb, which may have a low number of original annotations. Initially, the performance of network-based candidate protein prediction for each targeted anatomical entity is evaluated through leave-one-out cross-validation^28^, which facilitates the generation of ROC and precision-recall curves. Following this, a prediction precision threshold was used to discern new candidate proteins. A trial-and-error method was used to select the best precision threshold for each protein module, with the resultant module sizes taken into consideration. Modules for different precision thresholds were generated for pectoral fin-forelimb and pelvic fin-hindlimb entity pairs. The thresholds leading to the most comparable module sizes for aforementioned paired entities were selected. For example, a lower precision threshold was required for the pelvic fin due to the comparatively low number of original annotations.

Once the candidate proteins were predicted, respective modules for the pectoral fin and the pelvic fin were extracted from the zebrafish integrated network, and for the forelimb and hindlimb from the mouse network and visualized using Cytoscape software^39^.

### Validation of the predicted proteins

The validation process comprised three steps. Initially, the predicted proteins for the pectoral fin and pelvic fin modules in zebrafish were compared with the orthologous proteins in the forelimb and hindlimb modules in mouse and vice versa. This was to determine whether these proteins were annotated to a homologous anatomical entity. For example, if proteins predicted for the pectoral fin module appear in the forelimb module, this implies a certain degree of validation for those proteins based on homology.

The second step involved performing enrichment analyses to verify if the predicted proteins in each module share similar Biological Process terms from Gene Ontology (GO-BP) and Uberon annotations as the proteins with original annotations. The online functional enrichment analysis tool DAVID (https://david.ncifcrf.gov/) was used for gene/protein set enrichment analysis using GO-BP terms. DAVID uses Fisher’s exact test^40^ for enrichment analyses. Despite the common use of GO for enrichment analysis, anatomy ontologies are rarely used. To perform enrichment analysis using the Uberon anatomy ontology and Fisher’s exact test, a dedicated Python program (Uberon enrichment analysis program) was developed and used. Ontology terms with p-values less than 0.05 were considered as enriched terms.

In the third step, the weighted degree distributions of the predicted proteins were compared to the weighted degree distributions of the proteins with original annotations in each module. If the predicted proteins exhibit a higher weighted degree distribution, it suggests they hold similar or a greater importance as the proteins with original annotations. The degree of a protein is usually used to rank proteins and identify hub proteins. However, in weighted network analysis like the integrated networks used here, weighted degree is preferred over simple degree as it takes into account different interaction weights (Eq. 5) instead of merely counting the number of interactions for a particular node^26^.

5 $$Weighted\, degree = \mathop \sum \limits_{v \in n\left( u \right)} sim(v,u)$$

In Eq. 5, *n(u)* is the neighborhood of the protein of interest (*u*) and *v* iterates through all the neighbors of protein *u*. The interaction weight is denoted by *sim(v,u)*, which represents the protein similarity score for the interaction between proteins *v* and *u*. The weighted degree of protein *u* is derived from the sum of all interaction weights between protein *u* and all its neighbors.

### Comparison of the network modules

To identify the modular changes, the pectoral fin and pelvic fin modules of the zebrafish were compared with the forelimb and hindlimb modules of the mouse, respectively.

Because a whole genome duplication event occurred at the origin of actinopterygian fishes^41^, most mouse proteins have duplicated counterparts in zebrafish. To facilitate the module comparison, gene/protein ortholog mappings between mouse and zebrafish were retrieved from the Zebrafish Information Network^42^ (ZFIN; 06/26/2018; https://zfin.org/downloads). If a single mouse protein corresponded to multiple zebrafish orthologs within a zebrafish module, all zebrafish orthologs were retained. Through the module comparison, three categories of proteins were identified: conserved proteins (those common to two modules), zebrafish module-specific proteins, and mouse module-specific proteins. The weighted degree of each protein was calculated (Eq. 5) to identify the important hub proteins of each module, with the proteins then being ranked accordingly. The weighted degree of each zebrafish module protein was compared with the corresponding mouse ortholog to identify changes in importance during the transition. However, due to the differing sizes of the zebrafish and mouse modules, it was necessary to normalize each protein’s weighted degree by the total number of proteins in each module. Consequently, the normalized weighted degree distributions for conserved proteins, zebrafish module-specific proteins, and mouse module-specific proteins were compared for pectoral fin *versus* forelimb and pelvic fin *versus* hindlimb, thereby examining the relative importance of proteins within each group. Furthermore, Wilcoxon rank-sum tests were conducted on normalized weighted degree distributions between conserved and module-specific proteins for each fin and limb. This provided a statistical validation when comparing the importance of proteins in each group.

The fate of the zebrafish module-specific proteins in mouse was investigated by extracting mouse orthologs for the pectoral and pelvic fin module-specific proteins and performing enrichment analyses using Uberon and GO-BP terms. Similarly, the roles of the mouse module-specific proteins in zebrafish were investigated using zebrafish orthologs for the forelimb and hindlimb module-specific proteins. The DAVID online functional enrichment analysis tool was used to conduct gene/protein set enrichment analysis using GO-BP terms. Ontology terms with p-values less than 0.05 were considered as enriched terms.

## Results

### Detection of network modules

The zebrafish integrated network used to detect paired fin modules contained 17,394 proteins and 730,855 interactions, while the mouse integrated network used to identify paired limb modules encompassed 18,002 proteins and 613,671 interactions^28^. A breakdown of the number of proteins originally annotated to each anatomical entity is provided in Supplementary Table S1. The total number of proteins for the pectoral fin (198) and the forelimb (267) showed closer similarity compared to the total number of proteins for the pelvic fin (15) and the hindlimb (777).

The ROC and precision-recall curves created during the evaluation of network-based candidate protein predictions for each anatomical entity were provided in Supplementary Figs. S1 and S2, respectively. The curves indicate high accuracy for network-based candidate protein predictions for all anatomical entities (the AUC values of ROC curves were higher than 0.85), excluding the pelvic fin. This validates the high accuracy of the network-based candidate protein predictions, primarily owing to the integration of high-quality enhanced protein networks from our previous research^28^. The pelvic fin’s relatively lower performance might be attributed to a reduced number of original protein annotations. It is well established that prediction accuracy tends to improve when the size of the dataset or the number of protein annotations increases^43^. Anatomical entities with fewer protein annotations might yield lower AUC values.

The statistics for the extracted protein modules are given in Supplementary Table S1. Proteins with original annotations that were lost during the module extraction are listed in Supplementary Table S2. A high precision threshold of 0.7 was used for candidate protein predictions for pectoral fin, forelimb, and hindlimb modules. For the pelvic fin module, the precision threshold was reduced to 0.05 in order to achieve a comparable number of proteins to those in the hindlimb module.

Visual representations of the final modules for the pectoral fin, pelvic fin, forelimb, and hindlimb (paired fin and limb modules) are depicted in Supplementary Figs. S3, S4, S5, and S6, respectively. Supplementary Files S1, S2, S3, and S4 contain the accompanying Cytoscape network files for these modules. Proteins from the paired fin and limb modules, ranked based on the weighted degree, are listed in Supplementary Files S5, S6, S7, and S8, respectively.

### Validation of the predicted proteins

The lists of predicted proteins for paired fin and limb modules are provided in Supplementary Tables S3, S4, S5, and S6, respectively. From the 45 proteins predicted for the pectoral fin, 14 had mouse orthologs that were associated with the forelimb (9 direct annotations, 2 annotations specific to the parts or the developmental precursors, and 3 predicted). Out of the 605 proteins predicted for the pelvic fin, 78 had mouse orthologs related to the hindlimb (46 direct annotations, 20 annotations specific to the parts or the developmental precursors, and 12 predicted). From the 18 proteins predicted for the forelimb, 6 had zebrafish orthologs associated with the pectoral fin (2 direct annotations, 1 annotation solely to the parts or the developmental precursors, and 3 predicted). Finally, of 32 proteins predicted for the hindlimb, 12 had zebrafish orthologs connected to the pelvic fin (all 12 being predicted). These findings suggest that the orthologs of the predicted proteins are annotated to corresponding homologous anatomical entities, which adds a layer of validation to these predictions.

The shared enriched GO-BP terms among predicted proteins and proteins originally annotated for the paired fin and limb modules are enumerated in Supplementary Tables S7, S8, S9, and S10. Similarly, Supplementary Tables S11, S12, S13, and S14 outline the enriched Uberon terms common to the predicted proteins and proteins originally annotated for the paired fin and limb modules. Several fin/limb related GO-BP terms were commonly enriched across all modules, e.g., “pectoral fin development”, as were Uberon terms, such as “median fin fold”.

**Figure 2:**
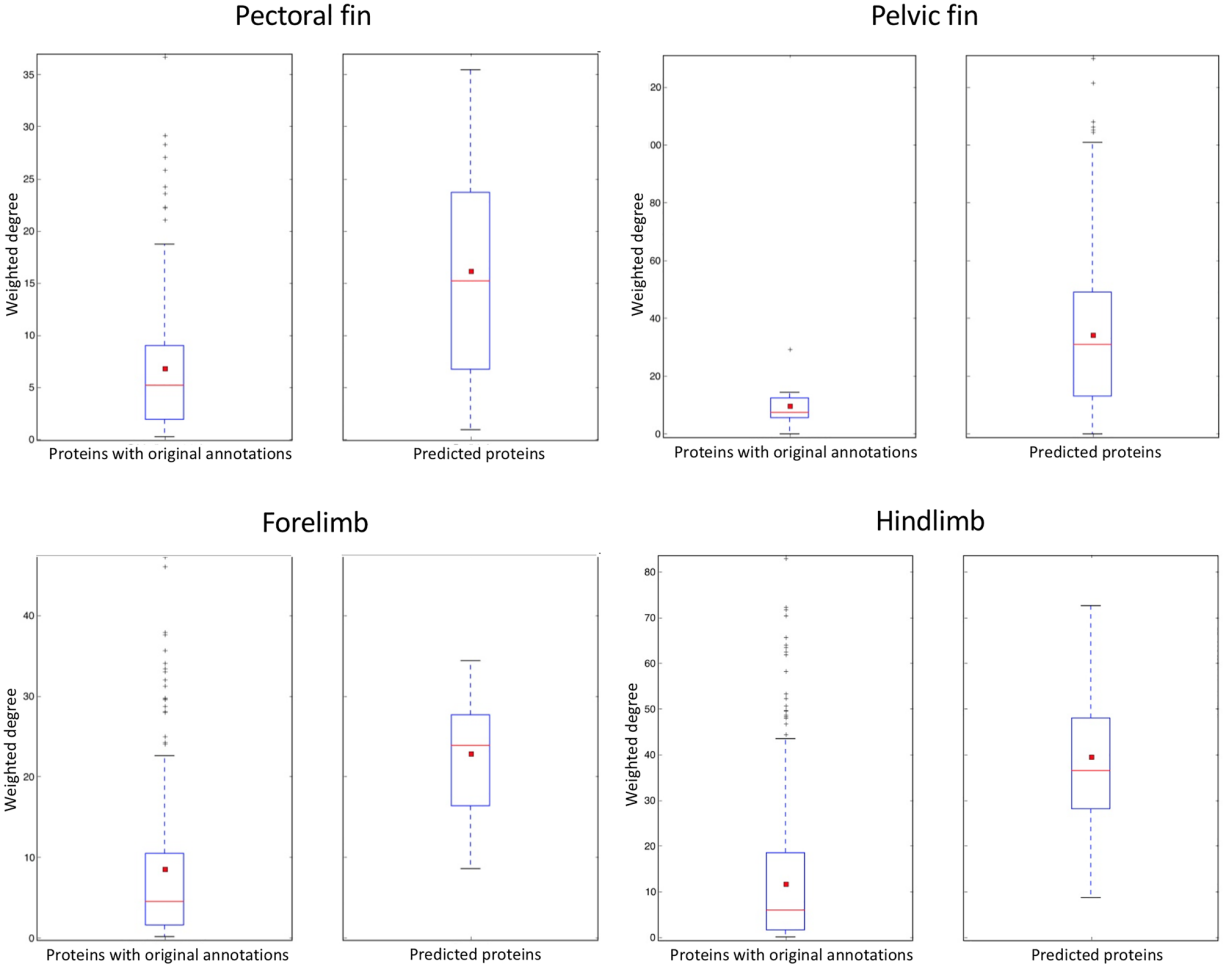
The boxplot comparisons of the weighted degree distributions for the predicted proteins *versus* proteins with original annotations for each module. The red line and the square represent the median and mean, respectively.

Boxplot comparisons (Fig. 2) show that across all paired fin and limb modules, the weighted degree distributions of predicted proteins exceeded those of proteins with original annotations. This pattern indicates that, as a collective, predicted proteins play central roles in module function.

### Comparison of the network modules

#### Pectoral fin vs. forelimb modules

**Figure 3:**
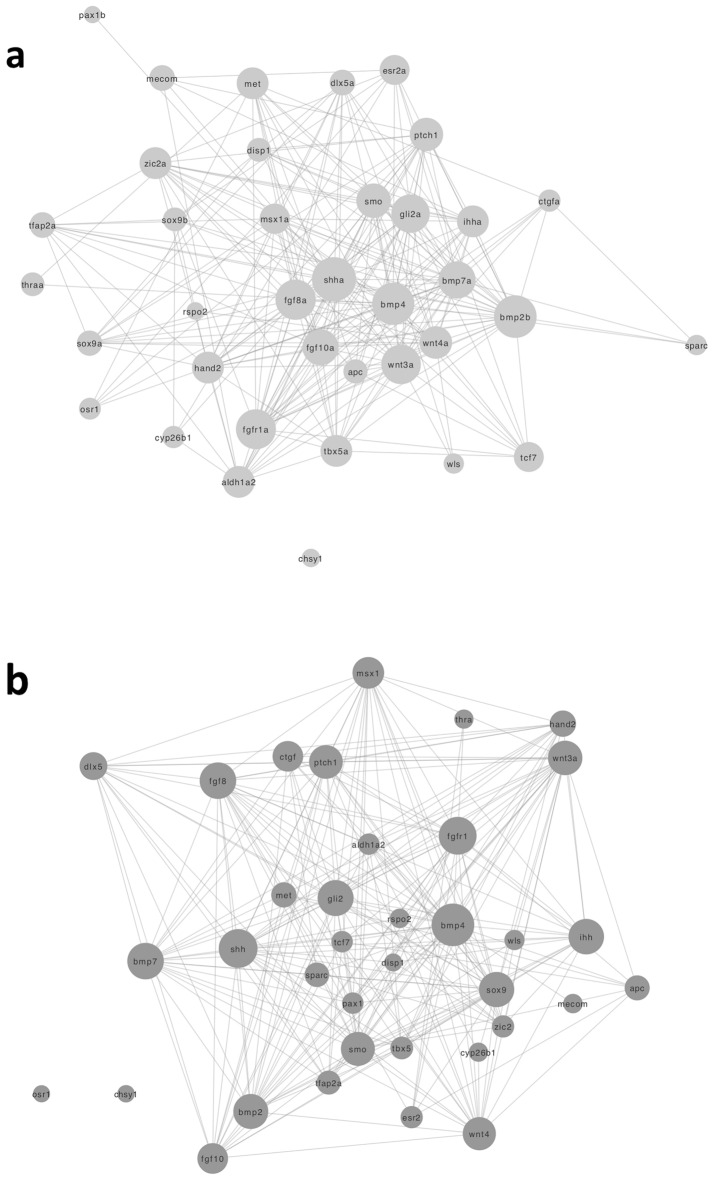
Network diagrams of the 37 conserved proteins shared between (**a**) the pectoral fin module and (**b**) the forelimb module. The size of each node is proportional to the degree (number of interactions) of the protein. Hub proteins, including *bmp4*, *shh*, *smo*, *bmp7, sox9*, and *gli2*, are represented by larger node sizes due to their high degrees of interaction.

A comparison of the proteins between these homologous structures yielded 183 proteins to be specific to the pectoral fin module, 207 proteins that were unique to the forelimb module, and 37 proteins shared (conserved) between both (Supplementary Table S15; Fig. 3).

In the pectoral fin module, the hub protein with the highest weighted degree was *shha* (sonic hedgehog a; Supplementary File S5), a protein known for its crucial role in pectoral fin development^16^. Its ortholog, *Shh*, ranked fourth in the forelimb module (Supplementary Table S15) and is widely studied for its role in the development and morphogenesis of limbs across species^44^, including mouse and humans. Notably, any disruptions in the sonic hedgehog signaling pathway in tetrapods correspond to losses, gains, or malformations of limbs^44^. The significance of *shh* gene in morphological patterning of both paired fins and limbs has made it a focal point in studies concerning the transition from fin to limb^12^.

The highest ranked protein in the forelimb module was *bmp4* (bone morphogenetic protein 4; Supplementary File S7). The *bmp4* plays a crucial role in the formation and morphogenesis of tetrapod limbs^45^. Mutations within *bmp4* can disrupt the *bmp4* signaling pathway, resulting in limb abnormalities in limb and digit formation^45^. The *bmp4* also held a high position in the pectoral fin module, ranking second, and was predicted during the module detection phase (Supplementary Table S15).

Of the conserved proteins (Fig. 3), some important hub proteins in pectoral fin module, such as *shha*, *bmp4*, *bmp2b*, and *bmp7a*, have retained their importance in forelimb development. This is underscored by their high rankings based on the weighted degree in the forelimb module (Supplementary Table S15). Other pectoral fin proteins, such as *sox9,* have a higher rank within the forelimb, reflecting a more substantial role in limb development. Within the pectoral fin module, the proteins *sox9a* and *sox9b* were ranked 83rd and 104th, respectively, whereas in mouse, the corresponding ortholog *sox9* was elevated to the 15th position (Supplementary Table S15). *Sox9* is renowned for its involvement in limb digit patterning, a process attributed to its role in the bmp-sox9-wnt Turing network^17^. Given that digits emerged after the transition from fins to limbs^12,15^, the involvement of *sox9* in a digit patterning pathway could have amplified the interactions it had with other proteins in the forelimb module, thereby increasing its importance.

**Figure 4:**
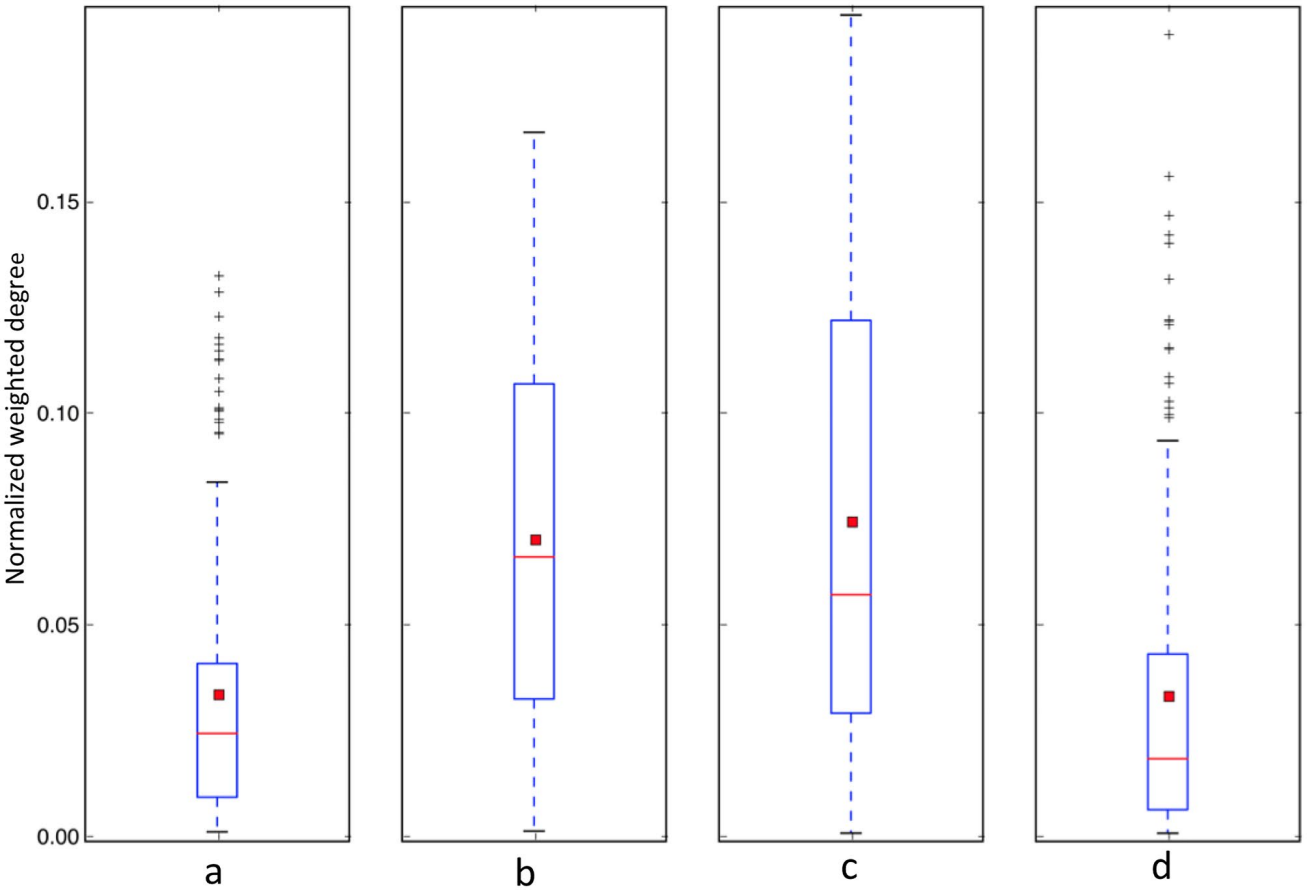
Boxplot comparison of normalized weighted degree distributions is presented for (**a**) pectoral fin module-specific proteins, (**b**) pectoral fin conserved proteins, (**c**) forelimb conserved proteins, and (**d**) forelimb module-specific proteins. The red line and square represent the median and mean, respectively.

A boxplot illustrating the normalized weighted degree distributions for pectoral fin module-specific proteins, pectoral fin conserved proteins (those shared with the forelimb), forelimb conserved proteins (those shared with the pectoral fin), and forelimb module-specific proteins is presented in Fig. 4. The conserved proteins in both modules exhibit higher normalized weighted degree distributions in comparison to their respective module-specific proteins (*p* = 5.97e−13 for pectoral fin and *p* = 1.69e−6 for forelimb) when comparing conserved versus module-specific proteins based on Wilcoxon rank-sum tests. This indicates that as a group, the conserved proteins engage in more interactions within the module and play a central role in maintaining modular stability. From an evolutionary point of view, it seems that during the transition from pectoral fin to the forelimb, proteins with higher degrees in the pectoral fin module, such as *shha* and *bmp4*, were retained in the forelimb. Moreover, new forelimb module-specific proteins were integrated into the forelimb, likely surrounding these conserved proteins.

#### Pelvic fin versus hindlimb modules

Comparisons revealed 536 specific proteins in the pelvic fin module, and 601 specific proteins in the hindlimb module. 81 proteins were conserved between the two modules (Supplementary Table S16 and Fig. S7).

In the pelvic fin module, the protein with the highest rank was *hsp90ab* (predicted; Supplementary File S6). Although *hsp90ab* is a heat shock protein and its impact on pelvic fin development is not well-established, its suppression has been linked to developmental defects in zebrafish, particularly in eye development^46^. Furthermore, the disruption of *hsp90ab* expression has been associated with caudal fin fold defects in zebrafish. This convergence of our computational findings and observed fin effects suggests that *hsp90ab* is a promising new candidate for pelvic fin development that may have a key role in the module stability.

The hub protein with the highest rank in the hindlimb module, *trp53*, is known to be associated with embryonic hindlimb development in mouse^47^. Although *trp53* also appears in the pelvic fin module (as a predicted protein), it held a lower rank (24th) based on the weighted degree (Supplementary Table S16).

Comparison of conserved proteins between pelvic fin and hindlimb modules (Supplementary Table S16 and Fig. S7) revealed several key proteins central to modular stability. For example, *ctnnb1*, a predicted module protein and ranked 4^th^ in the pelvic fin module, also held a high rank (3rd) in the hindlimb module. *Ctnnb1* is essential in the β-catenin pathway, which is necessary for mouse hindlimb initiation^48^. While there is as of yet no specific connection to zebrafish paired fins, *ctnnb1* is recognized as a critical element in overall fish development^49^.

According to comparison of normalized weighted degree distributions for pelvic fin module-specific proteins, pelvic fin conserved proteins, hindlimb conserved proteins, and hindlimb module-specific proteins (Supplementary Fig. S8), the conserved proteins in the hindlimb module demonstrated a higher normalized weighted degree distribution relative to the respective hindlimb module-specific proteins (*p* = 2.20e−16 based on the Wilcoxon rank-sum test). Meanwhile, the normalized weighted degree distribution for the pelvic fin module showed a moderate increase (*p* = 0.07, Wilcoxon rank-sum test). These findings underline the greater significance of conserved proteins in maintaining the stability of both the hindlimb and pelvic fin modules.

### The fate of zebrafish paired fin module-specific proteins in mouse

A large number of proteins found in zebrafish fin modules (83% for pectoral fin: 183 proteins; 80% for pelvic fin: 536 proteins) were not featured in the mouse limb modules (Supplementary Files S5 and S6), implying these proteins were not retained in limb development. To understand the roles of these proteins in mouse, the enriched GO-BP and Uberon terms for their mouse orthologs were investigated (Supplementary Tables S17, S18, S19 and S20). Several unique anatomical entities and corresponding biological processes exclusive to tetrapods^15^ were enriched (Supplementary Tables S21 and S22), such as the involvement of highly ranked (7th) pectoral fin module-specific protein *lef1* with palate development, trachea gland development, and neck-related phenotypes^50,51^.

### The role of mouse paired limb module-specific proteins in zebrafish

A majority of the limb module-specific proteins in mouse (85% for forelimb: 207 proteins; 90% for hindlimb: 601 proteins) did not appear in pectoral fin or pelvic fin modules in zebrafish (Supplementary Files S7 and S8). This suggests different roles for these proteins in zebrafish, prompting investigation into their enriched GO-BP and Uberon terms (Supplementary Tables S23, S24, S25 and S26). Some mouse limb module-specific proteins were enriched in the head region of the zebrafish, particularly in the jaw and post-hyoid pharyngeal arch skeleton (Supplementary Tables S27 and S28).

## Discussion

The application of PPI network methods enabled a deeper biomolecular exploration of the phenotypic transition from fin to limb and revealed new insights into this transition. A primary goal for this work was to identify hub proteins in the functional modules, and to compare them between fins and limbs. This study aimed to distinguish conserved and species-specific proteins in functional modules to better understand their roles in evolution. The results indicate that conserved proteins are more likely to be hub proteins than module-specific proteins, as evidenced by the weighted degree comparisons between these two groups (Fig. 4). This reinforces the initial hypothesis that during the fin-to-limb transition, most hub proteins from fin modules were preserved in limbs, with limb-specific proteins recruited to support this conserved appendage core network.

Furthermore, the results of this study imply that many proteins specific to the zebrafish fin modules were not retained in limb development. Instead, some appear to have been evolutionarily repurposed in the development of anatomical structures that emerged during the aquatic-to-terrestrial transition in vertebrates, such as the lungs and neck. Several unique anatomical entities and corresponding biological processes exclusive to tetrapods^15^ were enriched (Supplementary Tables S21 and S22) for fin module-specific proteins. For example, the highly ranked pectoral fin module-specific protein *lef1* is involved with palate development, trachea gland development, and neck-related phenotypes^50,51^. The evolution of the neck in tetrapods was instrumental in supporting the head, a critical adaptation for survival on land^52^. Similarly, the pelvic fin module-specific protein, *mapk1*, is associated with neck-related phenotypes, such as thymus development and trachea formation^53^. Additionally, it participates in lung development and other lung phenotypes^53^. Like the neck, the evolution of lungs in tetrapods was a significant factor enabling them to breathe and flourish in terrestrial environments^54^. Moreover, *Lama5*, a module-specific protein found in both pectoral fin and pelvic fin modules, is involved in mouse lung development^55^, hair follicle development, and hair-related phenotypes^56^, of which the latter anatomical features are unique to mammals^57^. These examples suggest that many proteins that initially played roles in fin development were co-opted for the development of novel anatomical structures, a move that helped tetrapods adapt to and thrive in terrestrial environments.

In addition, this research suggests that the original function of some specific proteins in mouse limb modules may have been in the development of non-paired fin structures, such as gill rakers and the caudal fin, which were gradually lost during the evolution of tetrapods. Investigation into their different potential roles in zebrafish showed that some mouse limb module-specific proteins were enriched in the head region of the zebrafish, particularly in the jaw and post-hyoid pharyngeal arch skeleton (Supplementary Tables S27 and S28). The latter encompasses the gill chamber and contains anatomical parts such as gill rakers^58^, which were lost during the evolution of tetrapods. For instance, *fst*, a crucial forelimb module-specific protein (Supplementary File S7), has a zebrafish ortholog (*fsta*) associated with splanchnocranium^59^ and post-hyoid pharyngeal arch skeleton^60^. Similarly, *twist1*, a module-specific protein for both forelimb and hindlimb, has two zebrafish orthologs (*twist1a* and *twist1b*) implicated in pharyngeal system development^61^. These enrichment analyses suggest that proteins initially associated with fish-specific structures, such as gill arches and the caudal fin, might have been co-opted for limb development as these structures were lost during the transition to tetrapods. These findings support Gegenbaur's theory, which proposes that pectoral and pelvic appendages in tetrapods originated from head branchial arches^62–64^. Despite initially being refuted and overshadowed in favor of the competing fin-fold theory, Gegenbaur's theory has seen a resurgence in support from recent evolutionary development (evo-devo) studies^58,62^. Together, these new findings and the generalized workflow developed here, sets the stage for further experimental exploration by evolutionary developmental biologists.

The network-based workflow used for this study presented several challenges and limitations which were mitigated using computational solutions. For instance, a single protein often plays a role in multiple phenotypes^3,4,28^; hence, it is crucial to verify whether the modular structure and underlying protein interactions are attributable exclusively to their involvement with the corresponding fins and limbs, or if they are associated with other phenotypes. Moreover, the presence of incorrect interactions in PPI networks can compromise the prediction accuracy of the modules^4,28^. To mitigate the confounding effects caused by other phenotypes and erroneous interactions, a network-based prediction model that has demonstrated a high degree of accuracy in our previous work^28^ was used. This model has been rigorously tested under various experimental conditions—including different semantic similarity calculation methodologies, various ontology annotation types, and multiple evaluation techniques—for both mouse and zebrafish, and has consistently delivered accurate candidate protein prediction results^28^. Another challenge faced during the study was that the module comparison depended on respective module sizes. For instance, the detection of the pelvic fin module presented challenges due to the low number of original proteins annotations, potentially because the pelvic fin bud arises late in development^65^ after gene disruptions may have killed the larval zebrafish. To address this issue, the prediction threshold for the pelvic fin module has to be lowered to extract a sizable module comparable to the hindlimb.

Generally, PPI networks retrieved from databases such as STRING are directly used for module detection^3,4^, but this work represents the inaugural application of integrated networks in addressing a biological problem. While the transition from fin to limb was the focal point of this study, this comparative integrative network-based approach could be applied to a range of other fields, such as human diseases, plant stress phenotypes, and more, paving the way for future directions in this line of research. In the future, this network comparison workflow could be applied, for example, to other significant evolutionary changes associated with aquatic-to-terrestrial vertebrate transition, such as changes in axial and cranial skeletons. Furthermore, a web-based application will be developed to easily compare the PPI network modules of any vertebrate anatomical change, which will enhance the usability of this workflow.

## Conclusions

This work represents the inaugural application of integrated networks in addressing a biological problem that may be generalized to many types of problems in comparative biology. This work enabled the identification of hub proteins essential to the anatomical transition from paired fins to limbs and an assessment of the hypothesis that hub proteins are most likely to be conserved during the transition. This approach also offered insights into the fate of fin module-specific proteins in terrestrial vertebrates, alongside the roles of limb module-specific proteins in fishes. Finally, this study presents a generalized network-based computational workflow designed to perform protein network module comparisons that can be more broadly used in investigating other evolutionary phenotypic transitions.

### Supplementary Information

Supplementary Figures.

Supplementary Tables.

Supplementary Information 1.

Supplementary Information 2.

Supplementary Information 3.

Supplementary Information 4.

Supplementary Information 5.

Supplementary Information 6.

Supplementary Information 7.

Supplementary Information 8.

Supplementary Information 9.

## Data Availability

The network files and anatomy profiles used for candidate protein predictions are available at: https://doi.org/10.6084/m9.figshare.13589579.v1. The additional figures and tables are included in electronic supplementary files.
