# Supplementary material for: Protein–protein interaction network module changes associated with the vertebrate fin-to-limb transition
Source: Sci Rep. 2023 Dec 18;13:22594. doi: 10.1038/s41598-023-50050-2 (PMC10730527; doi:10.1038/s41598-023-50050-2)
Supplement: Supplementary file 1 — Supplementary Figures. [file 41598_2023_50050_MOESM1_ESM.pdf]

Electronic Supplementary Figures

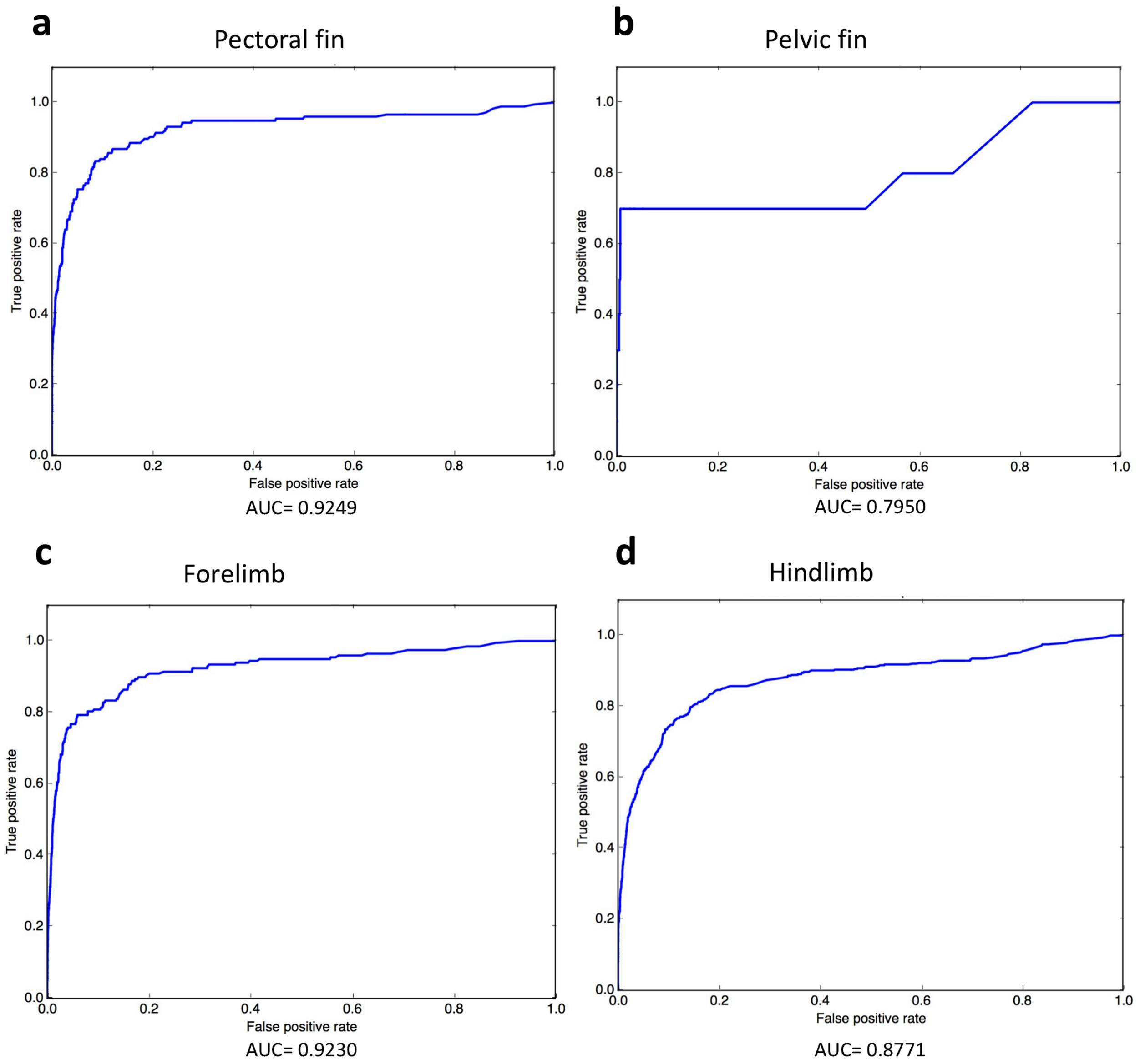

Supplementary Figure S1. The ROC curves for the four anatomical entities, including (a) Pectoral Fin, (b) Pelvic Fin, (c) Forelimb, (d) Hindlimb, generated during network-based candidate protein prediction evaluations.

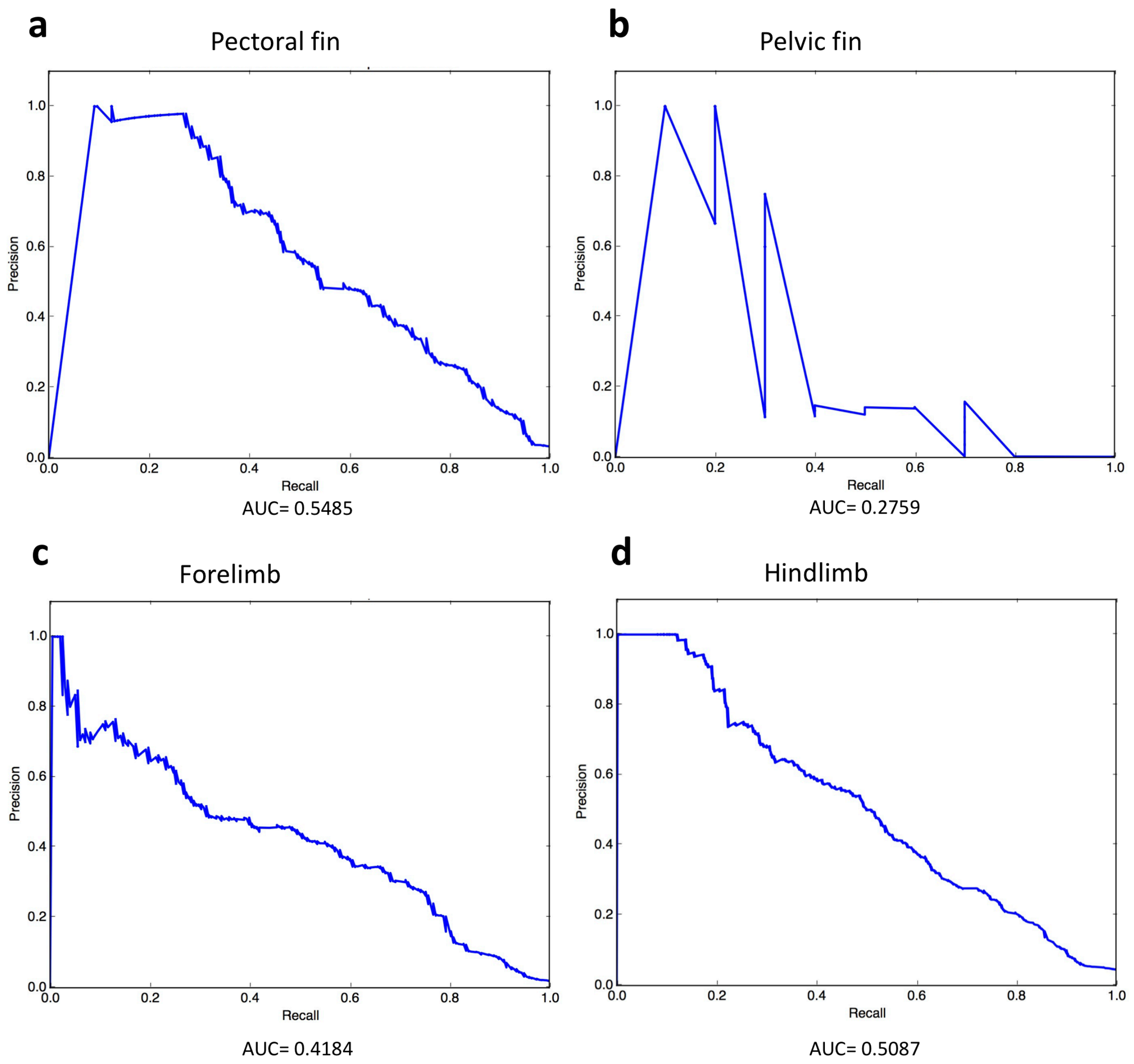

Supplementary Figure S2. The precision-recall curves for the four anatomical entities, including (a) Pectoral Fin, (b) Pelvic Fin, (c) Forelimb, (d) Hindlimb, generated during network-based candidate protein prediction evaluations.

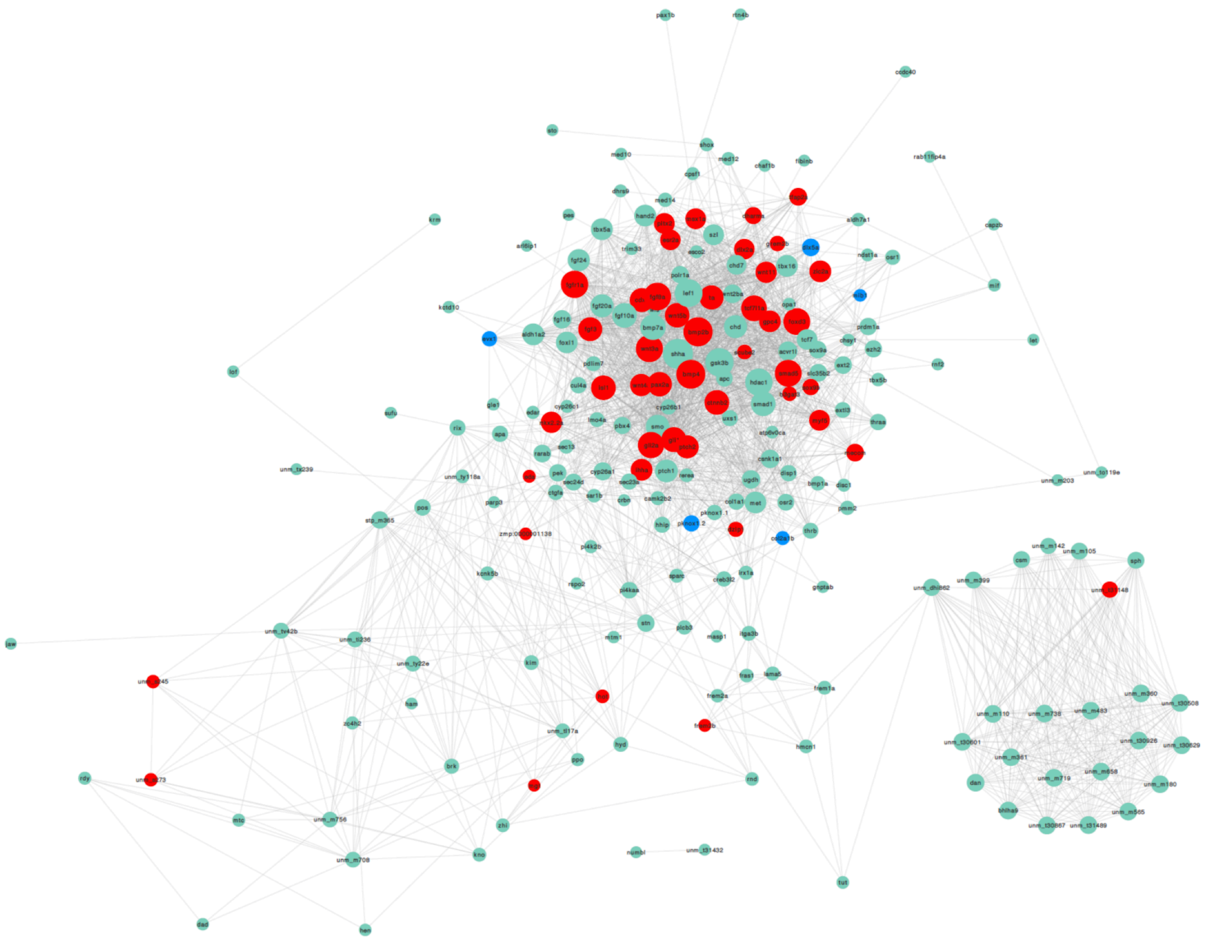

Supplementary Figure S3. Visualization of the pectoral fin module, including proteins with direct annotations to the pectoral fin (green), proteins annotated only to the pectoral fin parts or developmental precursors (blue), and predicted proteins (red). Node size is proportional to the degree (number of interactions) of the protein. An interactive version of this module is available in supplementary file S1 as a Cytoscape network file.

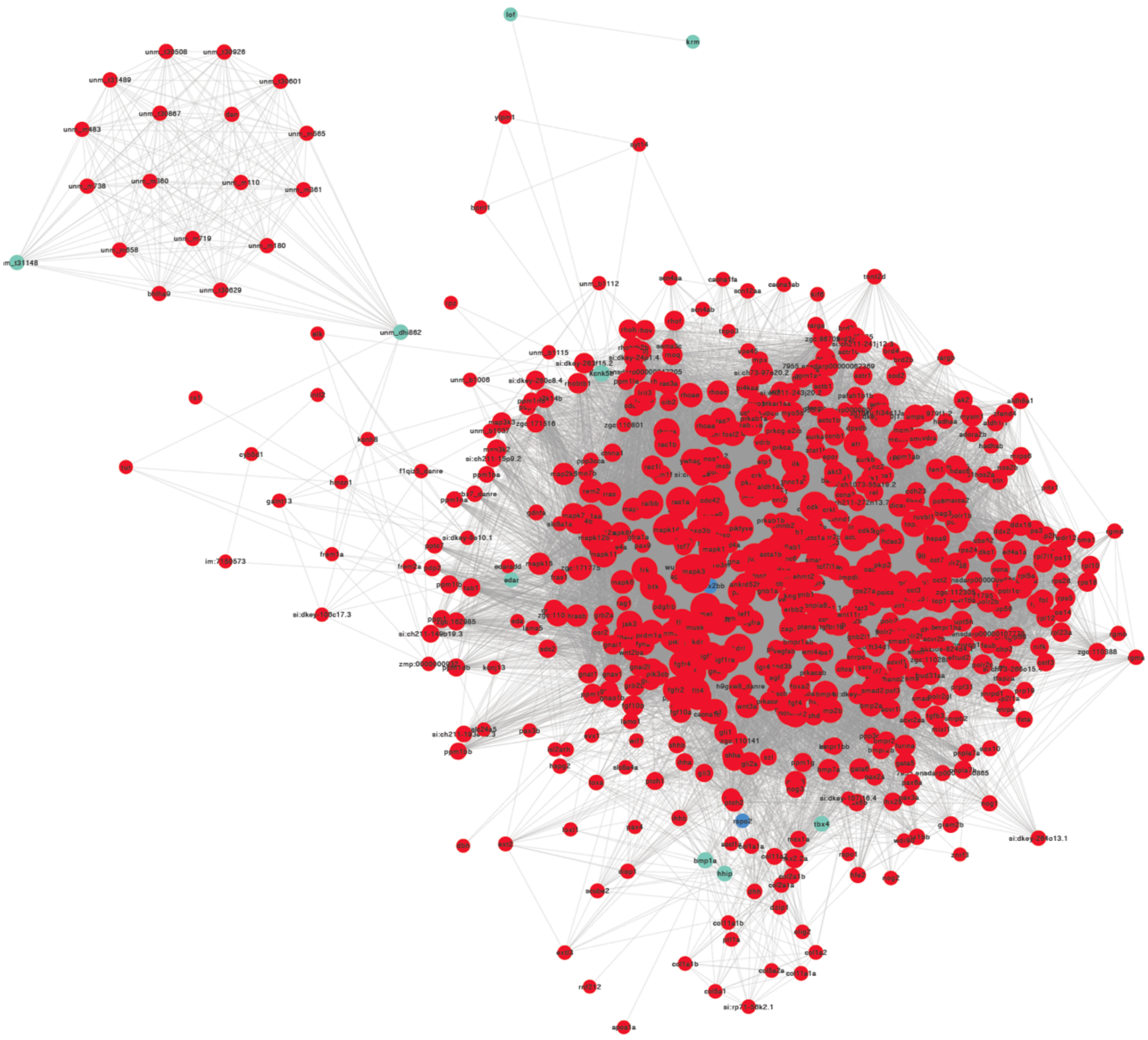

Supplementary Figure S4. Visualization of the pelvic fin module, including proteins with direct annotations to the pelvic fin (green), proteins annotated only to the pelvic fin parts or developmental precursors (blue), and predicted proteins (red). Node size is proportional to the degree (number of interactions) of the protein. An interactive version of this module is available in supplementary file S2 as a Cytoscape network file.

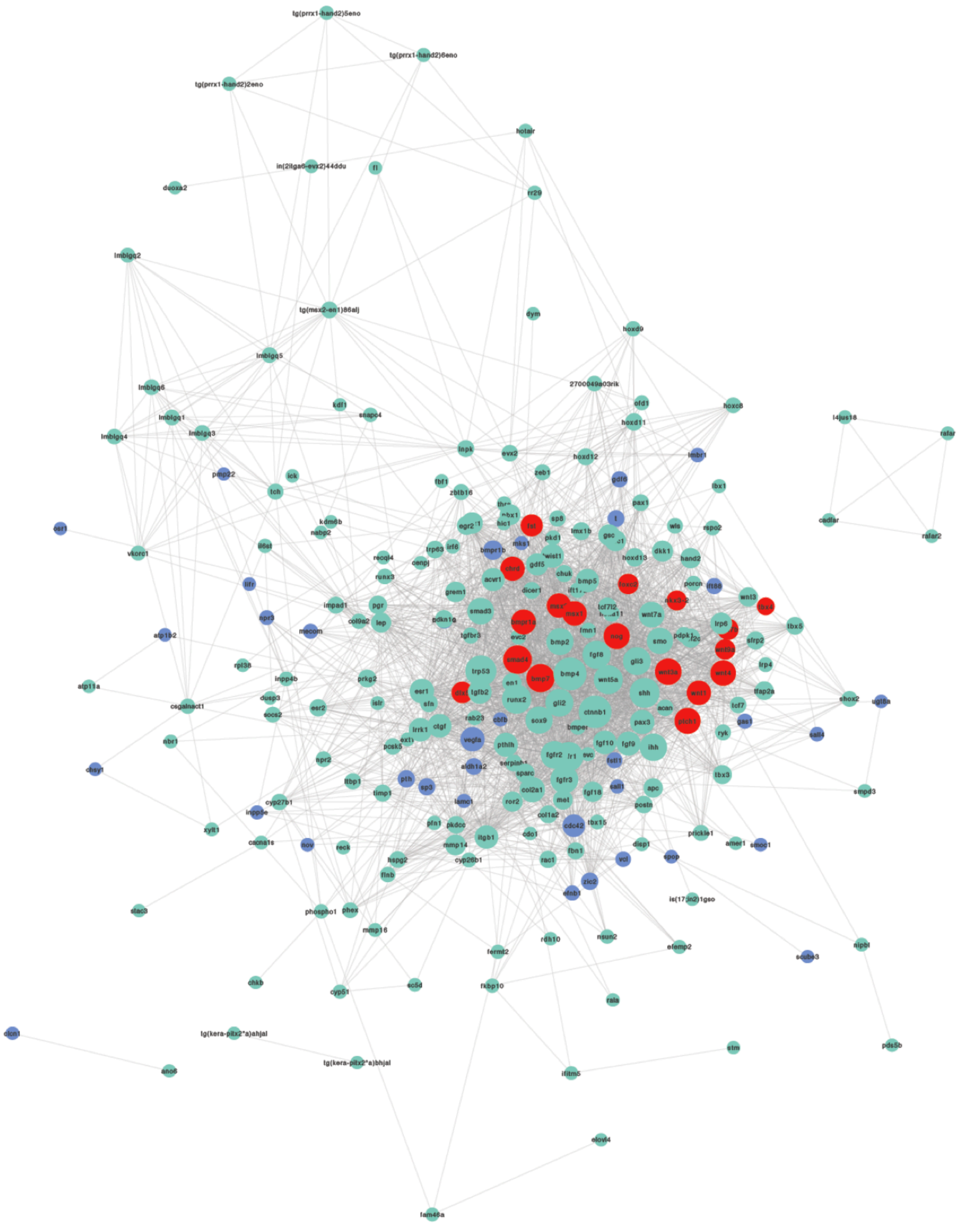

Supplementary Figure S5. Visualization of the forelimb module, including proteins with direct annotations to the forelimb (green), proteins annotated only to the forelimb parts or developmental precursors (blue), and predicted proteins (red). Node size is proportional to the degree (number of interactions) of the protein. An interactive version of this module is available in supplementary file S3 as a Cytoscape network file.

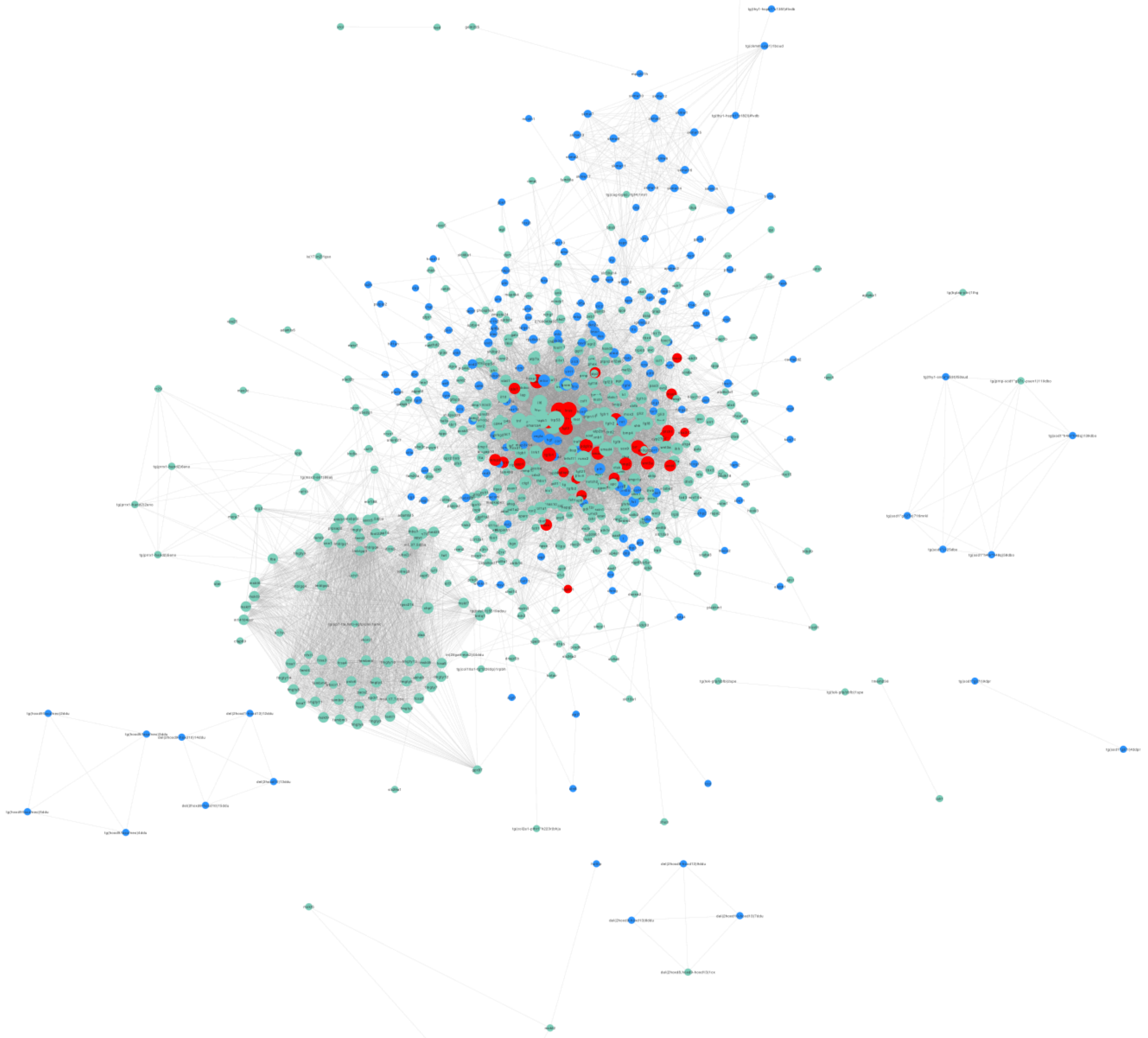

Supplementary Figure S6. Visualization of the hindlimb module, including proteins with direct annotations to the hindlimb (green), proteins annotated only to the hindlimb parts or developmental precursors (blue), and predicted proteins (red). Node size is proportional to the degree (number of interactions) of the protein. An interactive version of this module is available in supplementary file S4 as a Cytoscape network file.

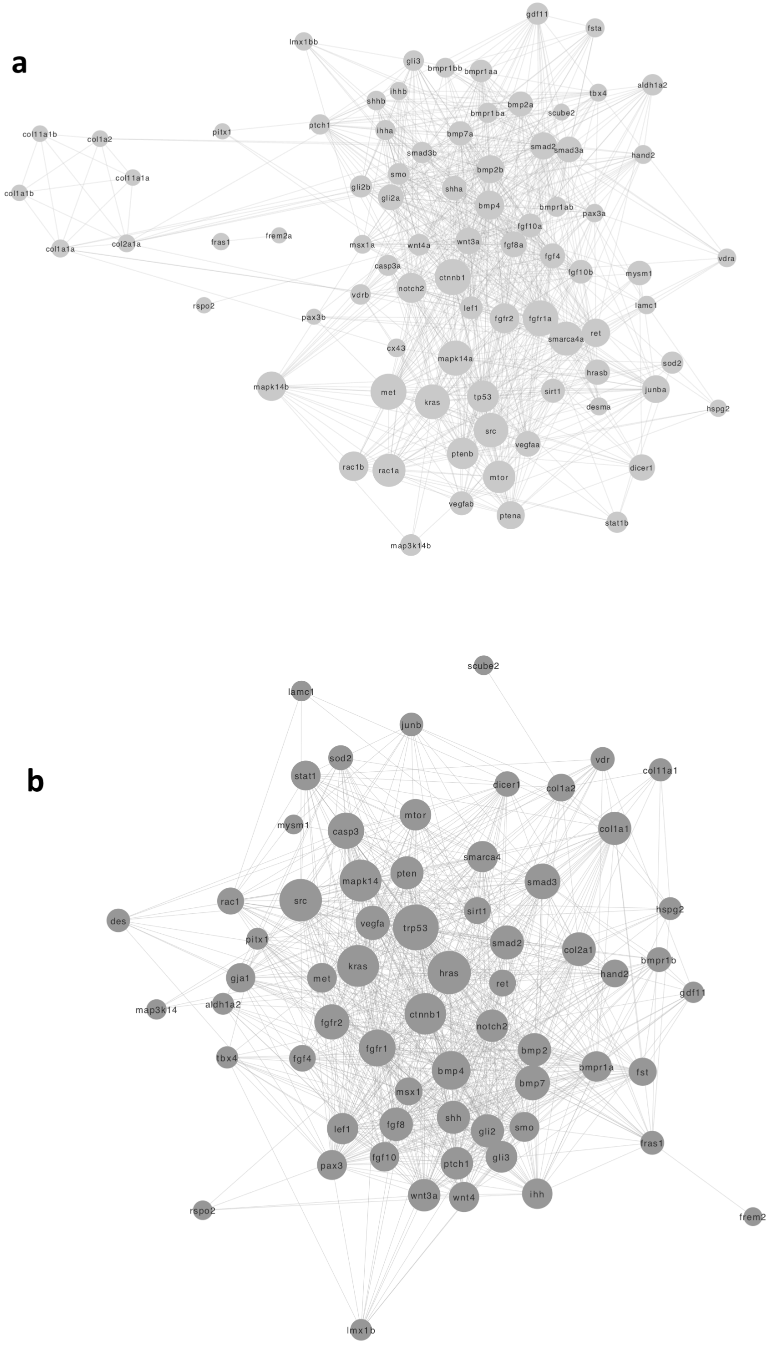

Supplementary Figure S7. Network diagrams of the 81 conserved proteins shared between (a) the pelvic fin module and (b) the hindlimb module. Node size is proportional to the degree (number of interactions) of the protein. Hub proteins, such as *bmp4*, *shh*, *ctnb1*, *bmp7, trp53*, and *hras*, are shown in larger node sizes.

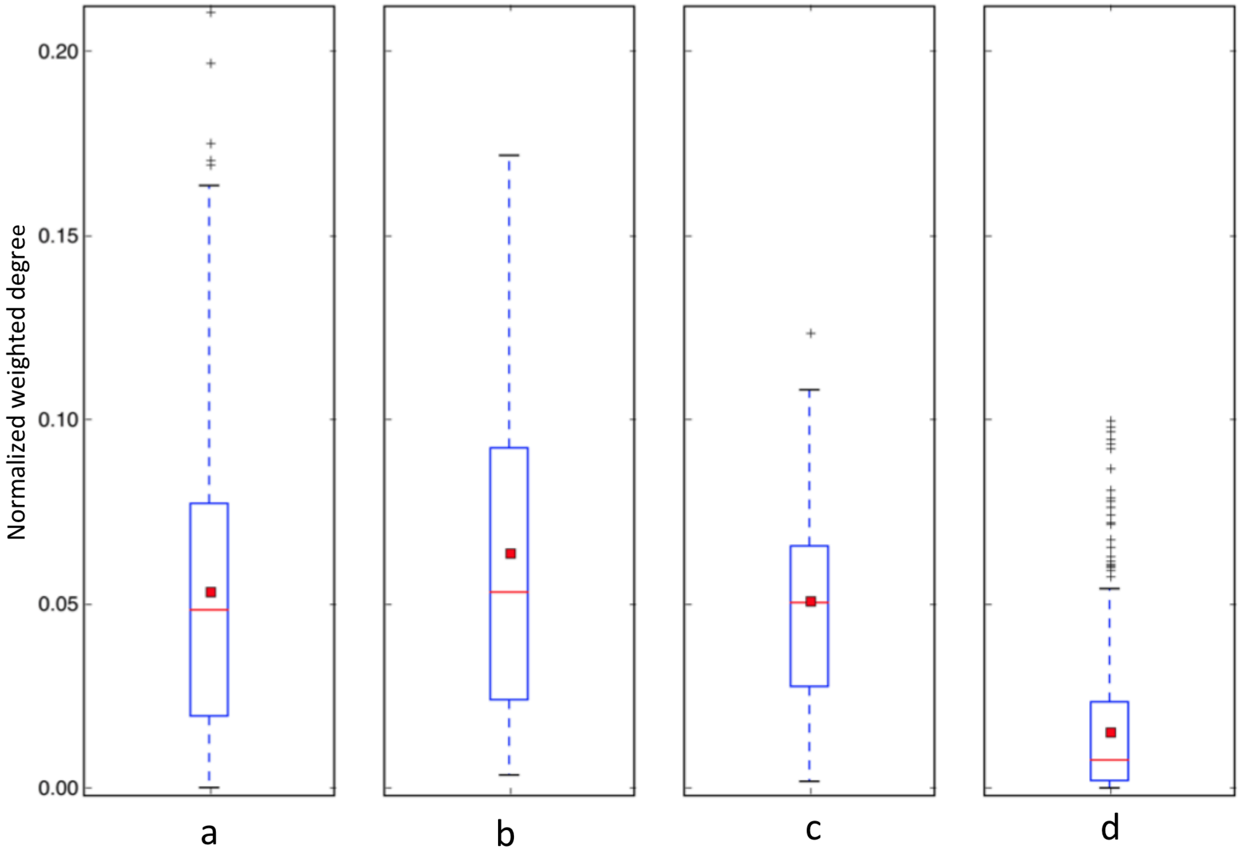

Supplementary Figure S8. Boxplot comparison of normalized weighted degree distributions for (a) pelvic fin module-specific proteins, (b) pelvic fin conserved proteins, (c) hindlimb conserved proteins, and (d) hindlimb module-specific proteins. The red line and square represent the median and mean, respectively.
