# Supplementary material for: Protein–protein interaction network module changes associated with the vertebrate fin-to-limb transition
Source: Sci Rep. 2023 Dec 18;13:22594. doi: 10.1038/s41598-023-50050-2 (PMC10730527; doi:10.1038/s41598-023-50050-2)
Supplement: Supplementary file 2 — Supplementary Tables. [file 41598_2023_50050_MOESM2_ESM.pdf]

Electronic Supplementary Tables

Supplementary Table S1. The statistics for anatomical entities and the extracted modules

	Pectoral fin	Pelvic fin	Forelimb	Hindlimb
Number of proteins with direct annotations to the anatomical entity	192	13	216	530
Number of proteins annotated only to the parts of the anatomical entity	3	2	44	239
Number of proteins annotated only to the bud of the anatomical entity	3	0	7	8
The total number of proteins used for the module detection	198	15	267	777
The precision threshold used for candidate protein prediction	0.7	0.05	0.7	0.7
Number of predicted proteins in the module	45	605	18	32
Number of proteins with original annotations to the anatomical entity, a part, or the bud in the module	175	12	225	639
Total number of proteins in the module	220	617	243	671
Number of proteins with original annotations that were lost due to the network cutoff	17	3	25	101

Number of proteins with original annotations that were lost due to isolation in the network	6	0	17	37

Supplementary Table S2. The proteins with original annotations that were lost due to network cutoff or isolation in the network

Pectoral fin	Pelvic fin	Forelimb	Hindlimb
**Lost due to network cutoff: 17**<br>1. *bcl2l16*<br>2. *bot*<br>3. *don*<br>4. *ful*<br>5. *gpatch3*<br>6. *hmx4*<br>7. *mko*<br>8. *not found*<br>9. *si:ch211-185a18.2*<br>10. *tmem260*<br>11. *unm au21*<br>12. *unm m572*<br>13. *unm ti205*<br>14. *unm tm136*<br>15. *unm to219*<br>16. *wan*<br>17. *zon*<br><br>**Lost due to isolation: 6**<br>1. *ap1g1*<br>2. *dul*<br>3. *gaz*<br>4. *nokr2*<br>5. *nrg2a*<br>6. *perp*	**Lost due to network cutoff: 3**<br>1. *mir223*<br>2. *sub*<br>3. *wan*<br><br>**Lost due to isolation: 0**	**Lost due to network cutoff: 25**<br>1. *am*<br>2. *ccd*<br>3. *cl*<br>4. *dbf*<br>5. *del(2hoxd1-hoxd10)26ddu*<br>6. *del(6dlx6-dlx5)1tlu*<br>7. *etn3*<br>8. *fts*<br>9. *is(in8b2-8b3.1;6c1)1tshir*<br>10. *lgl*<br>11. *mdga2*<br>12. *mhdaali18*<br>13. *mirc1*<br>14. *morc2a*<br>15. *os*<br>16. *pf*<br>17. *t(7;18)50h*<br>18. *tg(cag-mrfp1,-sox9,-egfp)1haak*<br>19. *tg(col2a1-mef2c/vp16)1eno*<br>20. *tg(hand2)#tshir*<br>21. *tg(pgk1-fgf2)15cofn*<br>22. *tg(plp1-lmnb1)1108qsp*<br>23. *tg(prrx1-sox9,-lacz)1haak*<br>24. *tg(tyr,col2a1-trpv4*r594h)#dhco*<br>25. *vsd*<br><br>**Lost due to isolation: 17**<br>1. *btd*<br>2. *cdk20*	**Lost due to network cutoff: 101**<br>1. *4933430i17rik*<br>2. *ano5*<br>3. *aspb*<br>4. *b2b1594clo*<br>5. *b2b2187clo*<br>6. *bolt*<br>7. *cby*<br>8. *cl*<br>9. *clec11a*<br>10. *dbf*<br>11. *del(6dlx6-dlx5)1tlu*<br>12. *dh*<br>13. *dmpy*<br>14. *dp(16cbr1-fam3b)1rhr*<br>15. *fts*<br>16. *gnd*<br>17. *hacd1*<br>18. *hxd*<br>19. *hydro*<br>20. *igf1sl1*<br>21. *inad*<br>22. *is(in8b2-8b3.1;6c1)1tshir*<br>23. *klhl41*<br>24. *lgl*<br>25. *lx*<br>26. *lz*<br>27. *map3k20*<br>28. *mhdaali18*<br>29. *mir140*<br>30. *mir92-1*<br>31. *mirc1*<br>32. *mpc234h*<br>33. *nad*<br>34. *nma*

		3. cpox<br>4. del(5d5mit73-d5mit351)5jcs<br>5. lgi4<br>6. nkx6-1<br>7. npat<br>8. ostm1<br>9. pappa2<br>10. pex10<br>11. rnf165<br>12. scx<br>13. tg(sod1*g127x)716mrkl<br>14. tram2<br>15. trpv4<br>16. uchl1<br>17. vps54	35. nmf419<br>36. not found<br>37. ogfod1<br>38. os<br>39. pf<br>40. pl<br>41. pma<br>42. ps<br>43. scarf2<br>44. skc3<br>45. srn<br>46. ssq<br>47. t(7;18)50h<br>48. tal<br>49. tenm4<br>50. tg(acta1-il15*)11650lsq<br>51. tg(ar*100q)c25als<br>52. tg(ar*100q)c32als<br>53. tg(b19-rnai:il3)241ckn<br>54. tg(cag-dsred2/rnai:tardbp)6zxu<br>55. tg(cd4-npm/alk)n1ingh<br>56. tg(ckmm-cav3)1ysu<br>57. tg(cnp-gpr17)1qrlu<br>58. tg(col11a2-npr2*)28keoz<br>59. tg(col1a1-ifitm5*)1brle<br>60. tg(col1a1)73prc<br>61. tg(ctnnb1)1efu<br>62. tg(eef1a1-gnas*r201c)184pabi<br>63. tg(epo*)458mym<br>64. tg(gfap-il6)g167lms<br>65. tg(gfap-il6)g16lms<br>66. tg(gfap-il6)g369lms<br>67. tg(h2-k-fosl1)1wag<br>68. tg(igkv3-5*-myc)#plbe<br>69. tg(lckil4)1315dbl<br>70. tg(mbp-cadm4*)#pele<br>71. tg(mt1-hgfsf)19lmb<br>72. tg(mt2a-tgfbr2)4rser<br>73. tg(myh7-pln)2egk<br>74. tg(nefl*e397k)#milg<br>75. tg(pgk1-fgf2)15cofn<br>76. tg(plp1*)4rsj<br>77. tg(prnp-ar*112q)#deme

				78. *tg(prnp-fus)wt3cshw*<br>79. *tg(prnp-mapt*p301l)jnpl3hlmc*<br>80. *tg(prnp-snca*a53t)83vle*<br>81. *tg(prnp-tardbp*a315t)23jlel*<br>82. *tg(prnp)c35cwe*<br>83. *tg(prnp*)#rgab*<br>84. *tg(s100b-v-erbb)4496waw*<br>85. *tg(sod1*)df7yaw*<br>86. *tg(sod1*g37r)106dpr*<br>87. *tg(sod1*g37r)29dpr*<br>88. *tg(sod1*g85r)148dwc*<br>89. *tg(sod1*g85r)74dwc*<br>90. *tg(sod1*g93a)<dl>1gur*<br>91. *tg(sod1*g93a)1gur*<br>92. *tg(sod1*h46r)lara*<br>93. *tg(tcrar28,tcrbr28)krndim*<br>94. *tg(teto-htr4*d100a)7niss*<br>95. *tg(thy1-mapt*p301l)2vln*<br>96. *tg(thy1-mapt*p301s)2541godt*<br>97. *tg(thy1-snca*a30p)18pjk*<br>98. *tg(thy1-ubqln2*p497s)3mont*<br>99. *tg(thy1-ubqln2*p506t)6mont*<br>100. *ts(17<16>)65dn*<br>101. *vsd*<br><br>**Lost due to isolation: 37**<br>1. *adgrf5*<br>2. *akap11*<br>3. *arid5b*<br>4. *col4a3bp*<br>5. *coq9*<br>6. *ctsf*<br>7. *dnase1l2*<br>8. *elmod1*<br>9. *epg5*<br>10. *hhipl1*<br>11. *hr*

			12. *lgi4*<br>13. *lncpint*<br>14. *ltn1*<br>15. *lyst*<br>16. *mbd5*<br>17. *noto*<br>18. *nxn*<br>19. *pex10*<br>20. *pgam5*<br>21. *pole4*<br>22. *psph*<br>23. *qk*<br>24. *rnf13*<br>25. *sacs*<br>26. *skida1*<br>27. *slc38a2*<br>28. *sno*<br>29. *spata6*<br>30. *spg20*<br>31. *stard10*<br>32. *tg(pmp22)c22clh*<br>33. *tg(prnp-fus*r521c)3313ejh*<br>34. *tg(sod1*g85r)#roos*<br>35. *tg(sod1*g86r)m3jwg*<br>36. *tg(thy1-mapt*)30schd*<br>37. *zfp106*

Supplementary Table S3. The 45 predicted proteins of the pectoral fin module ranked according to their weighted degrees.

Zebrafish protein name	ZFIN identifier	Weighted degree	Rank in the module	Mouse ortholog name	Mouse ortholog status
bmp4	zdb-gene-980528-2059	35.5652	2	*bmp4*	direct annotation to the forelimb
bmp2b	zdb-gene-980526-474	34.3109	3	*bmp2*	direct annotation to the forelimb
wnt3a	zdb-gene-001106-1	31.4927	4	*wnt3a*	predicted
fgf8a	zdb-gene-990415-72	31.0094	5	*fgf8*	direct annotation to the forelimb
gli2a	zdb-gene-990706-8	27.2862	8	*gli2*	direct annotation to the forelimb
wnt5b	zdb-gene-980526-87	25.9169	10	*wnt5b*	not associated with the forelimb
fgfr1a	zdb-gene-980526-255	25.6442	12	*fgfr1*	direct annotation to the forelimb
smad5	zdb-gene-990603-9	25.5690	13	*smad5*	not associated with the forelimb
gli1	zdb-gene-030321-1	25.2858	14	*gli1*	not associated with the forelimb
tcf7l1a	zdb-gene-980605-30	24.8101	15	*tcf7l1*	not associated with the forelimb
foxd3	zdb-gene-980526-143	24.7462	16	*foxd3*	not associated with the forelimb
ta	zdb-gene-980526-437	23.7992	18	*NA*	not associated with the forelimb
cdx4	zdb-gene-980526-330	23.1331	20	*cdx4*	not associated with the forelimb
pax2a	zdb-gene-990415-8	22.2653	22	*pax2*	not associated with the forelimb
ctnnb2	zdb-gene-040426-2575	22.1606	24	*NA*	not associated with the forelimb
ptch2	zdb-gene-980526-44	21.7001	25	*ptch2*	not associated with the forelimb
isl1	zdb-gene-980526-112	21.5418	26	*isl1*	not associated with the forelimb
fgf3	zdb-gene-980526-178	20.9767	28	*fgf3*	not associated with the forelimb
wnt4a	zdb-gene-980526-352	20.4183	29	*wnt4*	predicted
gpc4	zdb-gene-011119-1	18.5224	31	*gpc4*	not associated with the forelimb
ihha	zdb-gene-051010-1	18.1064	32	*ihh*	direct annotation to the forelimb
wnt11	zdb-gene-990603-12	17.1389	34	*wnt11*	not associated with the forelimb
zic2a	zdb-gene-000710-4	15.2976	40	*zic2*	annotated to a part or bud
nkx2.2a	zdb-gene-980526-403	15.0866	41	*nkx2-2*	not associated with the forelimb
dlx2a	zdb-gene-980526-212	14.8123	43	*dlx2*	not associated with the forelimb
pitx2	zdb-gene-990714-27	14.7898	44	*pitx2*	not associated with the forelimb

msx1a	zdb-gene-980526-312	14.6308	45	*msx1*	predicted
myf5	zdb-gene-000616-6	13.7077	48	*myf5*	not associated with the forelimb
esr2a	zdb-gene-030116-2	13.2000	51	*esr2*	direct annotation to the forelimb
dharma	zdb-gene-990415-22	9.6753	59	*NA*	not associated with the forelimb
tfap2a	zdb-gene-011212-6	9.2007	62	*tfap2a*	direct annotation to the forelimb
mecom	NA	8.4715	89	*mecom*	annotated to a part or bud
sox9b	zdb-gene-001103-2	7.2558	104	*sox9*	direct annotation to the forelimb
grem2b	zdb-gene-030911-9	6.8164	108	*grem2*	not associated with the forelimb
unm t31148	zdb-gene-070117-1894	6.6863	109	*NA*	not associated with the forelimb
dzip1	zdb-gene-040526-1	6.4409	112	*dzip1*	not associated with the forelimb
b3gat3	zdb-gene-020419-3	6.0316	115	*NA*	not associated with the forelimb
scube2	zdb-gene-050302-80	5.3349	123	*scube2*	not associated with the forelimb
hot	zdb-gene-070117-2108	2.7511	160	*NA*	not associated with the forelimb
frem2b	zdb-gene-081119-4	2.6947	163	*frem2*	not associated with the forelimb
unm s273	zdb-gene-070117-864	2.4459	167	*NA*	not associated with the forelimb
unm s245	zdb-gene-070117-865	2.4459	167	*NA*	not associated with the forelimb
eda	zdb-gene-050107-6	2.3936	169	*eda*	not associated with the forelimb
zmp:0000001138	zdb-gene-140106-98	2.1176	172	*NA*	not associated with the forelimb
mgt	zdb-gene-070117-2188	1.0103	197	*NA*	not associated with the forelimb

*NA indicates ‘not available’ due to the ortholog not found in the mouse

Supplementary Table S4. The top 50 predicted proteins of the pelvic fin module ranked according to their weighted degrees. The full predicted protein list is available on electronic material, Supplementary File S9.

Zebrafish protein name	ZFIN identifier	Weighted degree	Rank in the module	Mouse ortholog name	Mouse ortholog status
hsp90ab1	zdb-gene-990415-95	129.9415	1	*hsp90ab1*	not associated with the hindlimb
mapk3	zdb-gene-040121-1	121.5328	2	*mapk3*	not associated with the hindlimb
rhoab	zdb-gene-040322-2	108.0837	3	*rhoa*	not associated with the hindlimb
ctnnb1	zdb-gene-980526-362	106.2749	4	*ctnnb1*	direct annotation to the hindlimb
hsp90aa1.2	zdb-gene-031001-3	105.1273	5	*hsp90aa1*	not associated with the hindlimb
paics	zdb-gene-030131-9762	104.4687	6	*paics*	not associated with the hindlimb
gsk3b	zdb-gene-990714-4	101.2293	7	*gsk3b*	not associated with the hindlimb
cad	zdb-gene-021030-4	97.8373	8	*cad*	not associated with the hindlimb
cdc42	zdb-gene-030131-8783	97.7752	9	*NA*	not associated with the hindlimb
acta1b	zdb-gene-030131-55	97.6682	10	*acta1*	not associated with the hindlimb
smarca4a	zdb-gene-030605-1	97.4336	11	*smarca4*	direct annotation to the hindlimb
mapk1	zdb-gene-030722-2	96.3956	12	*mapk1*	not associated with the hindlimb
jupa	zdb-gene-991207-22	95.0796	13	*jup*	not associated with the hindlimb
cdk1	zdb-gene-010320-1	93.9169	14	*cdk1*	not associated with the hindlimb
kras	NA	93.4030	15	*kras*	predicted
rac1a	zdb-gene-030131-5415	92.9107	16	*rac1*	direct annotation to the hindlimb
mapk14a	zdb-gene-010202-2	92.1789	17	*mapk14*	direct annotation to the hindlimb
src	zdb-gene-030131-3809	91.7256	18	*src*	direct annotation to the hindlimb
fgfr1a	zdb-gene-980526-255	91.2553	19	*fgfr1*	direct annotation to the hindlimb
met	zdb-gene-041014-1	90.8735	20	*met*	direct annotation to the hindlimb
insrb	zdb-gene-020503-4	90.6827	21	*insr*	not associated with the hindlimb
si:ch211-163m16.1	NA	84.6841	22	*NA*	not associated with the hindlimb
actl6a	zdb-gene-020419-36	83.9023	23	*actl6a*	not associated with the hindlimb
tp53	zdb-gene-990415-270	83.8035	24	*trp53*	direct annotation to the hindlimb
pak2a	zdb-gene-021011-2	83.2637	25	*pak2*	not associated with the hindlimb
ehmt2	zdb-gene-010501-6	82.9897	26	*ehmt2*	not associated with the hindlimb
kdrl	zdb-gene-000705-1	81.0991	27	*NA*	not associated with the hindlimb
mtor	zdb-gene-030131-2974	80.8121	28	*mtor*	annotated to a part or bud
pkn2	zdb-gene-061207-42	79.5943	29	*pkn2*	not associated with the hindlimb
prkcbb	zdb-gene-040426-1178	79.5529	30	*prkcb*	not associated with the hindlimb
hsp90aa1.1	zdb-gene-990415-94	78.6325	31	*hsp90aa1*	not associated with the hindlimb

ptenb	zdb-gene-030616-47	78.5310	32	*pten*	annotated to a part or bud
kita	zdb-gene-980526-464	77.2229	33	*kit*	not associated with the hindlimb
akt2	zdb-gene-031007-5	77.0797	34	*akt2*	not associated with the hindlimb
igf1ra	zdb-gene-020503-1	76.5270	35	*igf1r*	not associated with the hindlimb
bmp4	zdb-gene-980528-2059	76.2150	36	*bmp4*	direct annotation to the hindlimb
igf1rb	zdb-gene-020503-2	75.0886	37	*igf1r*	not associated with the hindlimb
rap1b	zdb-gene-030131-9662	73.3502	38	*rap1b*	not associated with the hindlimb
rac2	zdb-gene-040625-27	73.3136	39	*rac2*	not associated with the hindlimb
hspa9	zdb-gene-030828-12	72.3324	40	*hspa9*	not associated with the hindlimb
hdac1	zdb-gene-020419-32	69.4428	41	*hdac1*	not associated with the hindlimb
pola1	zdb-gene-030114-9	69.0229	42	*pola1*	not associated with the hindlimb
actc1a	zdb-gene-040520-4	68.6027	43	*actc1*	not associated with the hindlimb
top2b	zdb-gene-041008-136	68.4413	44	*top2b*	not associated with the hindlimb
bmp2b	zdb-gene-980526-474	68.2429	45	*bmp2*	predicted
insra	zdb-gene-020503-3	68.1565	46	*insr*	not associated with the hindlimb
flt1	zdb-gene-050407-1	68.0761	47	*flt1*	not associated with the hindlimb
ralbb	zdb-gene-040625-121	67.8323	48	*ralb*	not associated with the hindlimb
btk	zdb-gene-070531-1	67.4762	49	*NA*	not associated with the hindlimb
rac1b	zdb-gene-060312-45	67.0359	50	*rac1*	direct annotation to the hindlimb

*NA indicates ‘not available’ due to the ortholog not found in the mouse

Supplementary Table S5. The 18 predicted proteins of the forelimb module ranked according to their weighted degrees.

Mouse protein name	MGI identifier	Weighted degree	Rank in the module	Zebrafish ortholog names	Zebrafish ortholog status
smad4	mgi:894293	34.5294	6	*smad4a, smad4b*	not associated with the pectoral fin
bmp7	mgi:103302	34.2132	7	*bmp7a*	direct annotation to the pectoral fin
wnt3a	mgi:98956	30.9124	14	*wnt3a*	predicted
nog	mgi:104327	29.3757	19	*NA*	not associated with the pectoral fin
wnt4	mgi:98957	27.9668	23	*wnt4a*	predicted
ptch1	mgi:105373	27.3374	24	*ptch1*	direct annotation to the pectoral fin
wnt1	mgi:98953	26.4361	25	*wnt1*	not associated with the pectoral fin
bmpr1a	mgi:1338938	26.0359	26	*bmpr1ab, bmpr1aa*	not associated with the pectoral fin
chrd	mgi:1313268	24.6081	28	*chrd*	not associated with the pectoral fin
msx1	mgi:97168	23.4172	31	*msx1a*	predicted
msx2	mgi:97169	22.8361	32	*msx2b, msx2a*	not associated with the pectoral fin
fst	mgi:95586	18.1062	46	*fsta, fstb*	not associated with the pectoral fin
wnt7b	mgi:98962	17.7777	47	*wnt7ba, wnt7bb*	not associated with the pectoral fin
dlx5	mgi:101926	16.1115	51	*dlx5a*	annotated to a part or bud
wnt9a	mgi:2446084	15.9927	52	*wnt9a*	not associated with the pectoral fin
foxc2	mgi:1347481	13.9395	61	*NA*	not associated with the pectoral fin
nkx3-2	mgi:108015	13.6063	63	*nkx3.2*	not associated with the pectoral fin
tbx4	mgi:102556	8.7400	84	*tbx4*	not associated with the pectoral fin

*NA indicates ‘not available’ due to the ortholog not found in the zebrafish

Supplementary Table S6. The 32 predicted proteins of the hindlimb module ranked according to their weighted degrees.

Mouse protein name	MGI identifier	Weighted degree	Rank in the module	Zebrafish ortholog names	Zebrafish ortholog status
hras	mgi:96224	72.8342	2	*hrasb*	predicted
kras	mgi:96680	70.7748	5	*kras*	predicted
myc	mgi:97250	66.8072	7	*mycb, myca*	not associated with the pelvic fin
fos	mgi:95574	64.7904	9	*fosab, fosaa*	not associated with the pelvic fin
tgfb1	mgi:98725	54.2763	15	*tgfb1b, tgfb1a*	not associated with the pelvic fin
igf1	mgi:96432	52.9341	17	*igf1*	not associated with the pelvic fin
fgf2	mgi:95516	51.2651	19	*fgf2*	not associated with the pelvic fin
casp3	mgi:107739	49.0774	23	*casp3a*	predicted
bmp7	mgi:103302	48.2316	26	*bmp7a*	predicted
wnt5a	mgi:98958	48.1588	27	*wnt5a*	not associated with the pelvic fin
smad2	mgi:108051	45.6514	30	*smad2*	predicted
pdgfra	mgi:97530	45.2074	31	*pdgfra*	not associated with the pelvic fin
bmp2	mgi:88177	45.0361	32	*bmp2a, bmp2b*	predicted
nog	mgi:104327	39.7063	46	*NA*	not associated with the pelvic fin
ptch1	mgi:105373	38.8191	48	*ptch1*	predicted
lef1	mgi:96770	36.7074	53	*lef1*	predicted
wnt1	mgi:98953	35.9468	60	*wnt1*	not associated with the pelvic fin
wnt4	mgi:98957	35.4014	62	*wnt4a*	predicted
mmp2	mgi:97009	35.3982	63	*mmp2*	not associated with the pelvic fin
spp1	mgi:98389	33.6944	70	*NA*	not associated with the pelvic fin
dcn	mgi:94872	33.0020	73	*dcn*	not associated with the pelvic fin
mmp14	mgi:101900	30.9303	77	*mmp14a, mmp14b*	not associated with the pelvic fin
chrd	mgi:1313268	29.8136	81	*chrd*	not associated with the pelvic fin
gja1	mgi:95713	28.3709	84	*cx43*	predicted
msx1	mgi:97168	26.6468	88	*msx1a*	predicted
twist1	mgi:98872	25.5266	93	*twist1a, twist1b*	not associated with the pelvic fin
bglap	mgi:88156	24.8621	99	*NA*	not associated with the pelvic fin
bglap2	mgi:88157	24.8447	100	*NA*	not associated with the pelvic fin
sp7	mgi:2153568	21.0093	119	*sp7*	not associated with the pelvic fin
foxc2	mgi:1347481	17.4167	192	*NA*	not associated with the pelvic fin
nkx3-2	mgi:108015	15.3314	207	*nkx3.2*	not associated with the pelvic fin

hapln1	mgi:1337006	8.9023	279	*hapln1a, hapln1b*	not associated with the pelvic fin

*NA indicates ‘not available’ due to the ortholog not found in the zebrafish

Supplementary Table S7. The enriched Biological Process terms from the Gene Ontology that are common to the predicted proteins and proteins with original annotations for the pectoral fin. The enriched terms are sorted based on the p-value of those terms for the predicted proteins.

Term identifier	Term name	P-value for predicted proteins	P-value for original proteins
GO:0007275	multicellular organism development	2.41E-12	1.85E-09
GO:0009953	dorsal/ventral pattern formation	6.32E-12	2.23E-06
GO:0051216	cartilage development	2.28E-11	4.70E-17
GO:0009880	embryonic pattern specification	4.19E-09	0.0160149
GO:0006355	regulation of transcription, DNA-templated	4.82E-09	1.70E-07
GO:0014032	neural crest cell development	2.91E-08	0.00016897
GO:0001756	somitoproteinsis	6.37E-08	2.80E-07
GO:0007368	determination of left/right symmetry	1.71E-07	4.78E-09
GO:0030182	neuron differentiation	3.70E-07	9.35E-05
GO:0007224	smoothened signaling pathway	6.03E-07	0.00117078
GO:0048703	embryonic viscerocranium morphoproteinsis	3.24E-06	2.95E-16
GO:2000223	regulation of BMP signaling pathway involved in heart jogging	3.31E-06	0.0037629
GO:0042476	odontoproteinsis	4.29E-06	2.35E-06
GO:0021984	adenohypophysis development	4.29E-06	0.00442453
GO:0030902	hindbrain development	4.52E-06	4.34E-08
GO:0009952	anterior/posterior pattern specification	7.59E-06	0.00060755
GO:0001947	heart looping	8.61E-06	6.61E-15
GO:0016055	Wnt signaling pathway	1.66E-05	6.53E-06
GO:0048793	pronephros development	2.04E-05	1.01E-05
GO:0003143	embryonic heart tube morphoproteinsis	3.81E-05	3.94E-08
GO:0031018	endocrine pancreas development	5.91E-05	0.02264448
GO:0042694	muscle cell fate specification	9.42E-05	0.04578302
GO:0031290	retinal ganglion cell axon guidance	0.000111737	9.33E-06
GO:0048701	embryonic cranial skeleton morphoproteinsis	0.000292195	5.06E-09
GO:0060070	canonical Wnt signaling pathway	0.00042765	8.61E-05
GO:0021508	floor plate formation	0.000564411	0.00513581

GO:0048264	determination of ventral identity	0.001287915	0.00055075
GO:0060037	pharyngeal system development	0.001287915	0.00055075
GO:0060041	retina development in camera-type eye	0.001442926	5.42E-06
GO:0071599	otic vesicle development	0.001684552	0.0148082
GO:0030917	midbrain-hindbrain boundary development	0.002132019	0.00117078
GO:0060059	embryonic retina morphoproteinsis in camera-type eye	0.002132019	0.01854958
GO:0045893	positive regulation of transcription, DNA-templated	0.002141963	0.04413722
GO:0008543	fibroblast growth factor receptor signaling pathway	0.003568028	0.03020605
GO:0031016	pancreas development	0.003981011	9.33E-06
GO:0001501	skeletal system development	0.003981011	0.00293934
GO:0043010	camera-type eye development	0.007171621	0.00060755
GO:0007507	heart development	0.010543118	1.44E-08
GO:0003342	proepicardium development	0.012678336	0.00059083
GO:0010002	cardioblast differentiation	0.022708189	3.72E-05
GO:0045892	negative regulation of transcription, DNA-templated	0.024331089	0.00642797
GO:0030166	proteoglycan biosynthetic process	0.025200109	0.00259184
GO:0021986	habenula development	0.032638807	0.00442453
GO:0048384	retinoic acid receptor signaling pathway	0.035106069	0.00015684
GO:0001649	osteoblast differentiation	0.035106069	0.00015684
GO:0048709	oligodendrocyte differentiation	0.035106069	3.28E-06
GO:0030198	extracellular matrix organization	0.047350757	0.00940935

Supplementary Table S8. The enriched Biological Process terms from the Gene Ontology that are common to the predicted proteins and proteins with original annotations for the pelvic fin. The enriched terms are sorted based on the p-value of those terms for the predicted proteins.

Term identifier	Term name	P-value for the predicted proteins	P-value for the original proteins
GO:0033333	fin development	7.11E-10	9.72E-08

Supplementary Table S9. The enriched Biological Process terms from the Gene Ontology that are common to the predicted proteins and proteins with original annotations for the forelimb. The enriched terms are sorted based on the p-value of those terms for the predicted proteins.

Term identifier	Term name	P-value for the predicted proteins	P-value for the original proteins
GO:0030509	BMP signaling pathway	2.12E-17	2.19E-10
GO:0007275	multicellular organism development	5.15E-12	5.81E-25
GO:0007389	pattern specification process	1.71E-09	1.90E-15
GO:0030326	embryonic limb morphoproteinsis	2.62E-09	1.44E-40
GO:0045669	positive regulation of osteoblast differentiation	3.87E-09	2.15E-10
GO:0045893	positive regulation of transcription, DNA-templated	1.91E-08	8.27E-22
GO:0001701	in utero embryonic development	2.06E-07	3.67E-14
GO:0042475	odontoproteinsis of dentin-containing tooth	3.08E-07	2.14E-16
GO:0042733	embryonic digit morphoproteinsis	3.72E-07	6.50E-38
GO:0051216	cartilage development	8.94E-07	1.05E-21
GO:0060021	palate development	1.03E-06	1.88E-18
GO:0060070	canonical Wnt signaling pathway	1.24E-06	1.80E-11
GO:0045944	positive regulation of transcription from RNA polymerase II promoter	1.27E-06	5.94E-32
GO:0000122	negative regulation of transcription from RNA polymerase II promoter	2.30E-06	3.42E-23
GO:0001501	skeletal system development	2.60E-06	9.14E-36
GO:0009952	anterior/posterior pattern specification	3.12E-06	1.71E-17
GO:0001649	osteoblast differentiation	3.23E-06	3.89E-10
GO:0001707	mesoderm formation	4.43E-06	4.00E-05
GO:0071773	cellular response to BMP stimulus	4.43E-06	4.00E-05
GO:0007411	axon guidance	9.70E-06	1.08E-07

GO:0001658	branching involved in ureteric bud morphoproteinsis	1.16E-05	3.84E-12
GO:0009953	dorsal/ventral pattern formation	1.24E-05	1.42E-21
GO:0008285	negative regulation of cell proliferation	2.11E-05	1.63E-13
GO:0003007	heart morphoproteinsis	2.78E-05	2.07E-09
GO:0090263	positive regulation of canonical Wnt signaling pathway	3.19E-05	8.99E-12
GO:0016055	Wnt signaling pathway	3.95E-05	2.85E-11
GO:0001503	ossification	9.34E-05	1.17E-21
GO:0090090	negative regulation of canonical Wnt signaling pathway	0.000111887	1.05E-10
GO:0009887	organ morphoproteinsis	0.000136246	1.77E-11
GO:0045892	negative regulation of transcription, DNA-templated	0.00014868	3.08E-08
GO:0045596	negative regulation of cell differentiation	0.000155607	0.00172739
GO:0048646	anatomical structure formation involved in morphoproteinsis	0.000226843	5.78E-06
GO:0042474	middle ear morphoproteinsis	0.000226843	5.78E-06
GO:0003148	outflow tract septum morphoproteinsis	0.000226843	0.000132268
GO:0001822	kidney development	0.000229367	1.74E-17
GO:0021983	pituitary gland development	0.00035634	1.84E-05
GO:0001837	epithelial to mesenchymal transition	0.000380704	0.00036855
GO:0060349	bone morphoproteinsis	0.00040586	4.87E-11
GO:0035115	embryonic forelimb morphoproteinsis	0.000514369	6.35E-37
GO:0030501	positive regulation of bone mineralization	0.000543461	1.62E-13
GO:0060325	face morphoproteinsis	0.000635432	7.74E-05
GO:0048568	embryonic organ development	0.000635432	4.91E-06
GO:0007492	endoderm development	0.000635432	0.000990141
GO:0030901	midbrain development	0.000635432	1.15E-08
GO:0030514	negative regulation of BMP signaling pathway	0.000840378	0.000152854
GO:0010862	positive regulation of pathway-restricted SMAD protein phosphorylation	0.000914879	1.46E-05
GO:0001657	ureteric bud development	0.000914879	9.46E-07
GO:0045599	negative regulation of fat cell differentiation	0.000914879	0.001967343
GO:0003151	outflow tract morphoproteinsis	0.001156789	7.48E-09
GO:0045668	negative regulation of osteoblast differentiation	0.001243528	0.00346626
GO:0001756	somitoproteinsis	0.001243528	1.82E-07
GO:0045165	cell fate commitment	0.002045776	2.19E-11
GO:0042472	inner ear morphoproteinsis	0.002045776	1.01E-07
GO:0050679	positive regulation of epithelial cell proliferation	0.002276056	1.05E-13
GO:0006355	regulation of transcription, DNA-templated	0.0030741	1.70E-09
GO:0007267	cell-cell signaling	0.004133083	1.62E-09

GO:0030336	negative regulation of cell migration	0.004371326	0.030620272
GO:0006351	transcription, DNA-templated	0.005714263	2.85E-08
GO:0030324	lung development	0.005931081	6.63E-18
GO:0048762	mesenchymal cell differentiation	0.010296133	0.00020907
GO:0060272	embryonic skeletal joint morphoproteinsis	0.01122718	1.20E-07
GO:0043616	keratinocyte proliferation	0.012157404	0.008847589
GO:0061053	somite development	0.013086804	0.00045
GO:0008284	positive regulation of cell proliferation	0.013313841	3.23E-18
GO:0014032	neural crest cell development	0.015870067	8.96E-07
GO:0003203	endocardial cushion morphoproteinsis	0.015870067	3.15E-05
GO:0035137	hindlimb morphoproteinsis	0.015870067	1.96E-08
GO:0032331	negative regulation of chondrocyte differentiation	0.016796179	2.91E-08
GO:0051145	smooth muscle cell differentiation	0.016796179	4.01E-05
GO:0060037	pharyngeal system development	0.016796179	0.016734602
GO:0048856	anatomical structure development	0.016796179	4.01E-05
GO:0021904	dorsal/ventral neural tube patterning	0.017721471	4.21E-08
GO:0008283	cell proliferation	0.017771413	3.67E-05
GO:0045879	negative regulation of smoothened signaling pathway	0.019569595	7.65E-05
GO:0023019	signal transduction involved in regulation of gene expression	0.021414447	0.002033958
GO:0060425	lung morphoproteinsis	0.022335647	0.002305628
GO:0007498	mesoderm development	0.0241756	0.002913859
GO:0007507	heart development	0.024473032	1.18E-18
GO:0042476	odontoproteinsis	0.025094353	4.27E-07
GO:0035108	limb morphoproteinsis	0.026012293	6.57E-17
GO:0001702	gastrulation with mouth forming second	0.026012293	0.038461084
GO:0071542	dopaminergic neuron differentiation	0.026929419	7.12E-10
GO:0048663	neuron fate commitment	0.029675922	2.55E-05
GO:0035116	embryonic hindlimb morphoproteinsis	0.030589801	1.87E-19
GO:0048589	developmental growth	0.032415128	1.19E-10
GO:0034504	protein localization to nucleus	0.032415128	0.000592382
GO:0048754	branching morphoproteinsis of an epithelial tube	0.033326579	4.55E-12
GO:0002053	positive regulation of mesenchymal cell proliferation	0.034237221	1.34E-18
GO:0030154	cell differentiation	0.034609309	1.49E-12
GO:0060412	ventricular septum morphoproteinsis	0.035147055	0.008576143
GO:0030879	mammary gland development	0.035147055	6.02E-05
GO:0001656	metanephros development	0.035147055	7.45E-09
GO:0033077	T cell differentiation in thymus	0.036056083	0.000898871

GO:0048706	embryonic skeletal system development	0.040589145	4.11E-11
GO:0045597	positive regulation of cell differentiation	0.042396746	1.13E-05
GO:0048705	skeletal system morphoproteinsis	0.049595145	1.36E-07

Supplementary Table S10. The enriched Biological Process terms from the Gene Ontology that are common to the predicted proteins and proteins with original annotations for the hindlimb. The enriched terms are sorted based on the p-value of those terms for the predicted proteins.

Term identifier	Term name	P-value for the predicted proteins	P-value for the original proteins
GO:0045944	positive regulation of transcription from RNA polymerase II promoter	4.34E-14	7.62E-37
GO:0045893	positive regulation of transcription, DNA-templated	3.71E-12	4.70E-31
GO:0001649	osteoblast differentiation	1.27E-11	5.39E-12
GO:0050679	positive regulation of epithelial cell proliferation	4.24E-11	2.50E-09
GO:0001837	epithelial to mesenchymal transition	1.74E-09	0.00157941
GO:0060325	face morphoproteinsis	6.66E-09	0.00497598
GO:0008285	negative regulation of cell proliferation	1.07E-08	1.18E-17
GO:0010629	negative regulation of gene expression	1.13E-08	7.93E-09
GO:0008284	positive regulation of cell proliferation	1.36E-08	8.36E-26
GO:0010628	positive regulation of gene expression	1.49E-08	5.42E-17
GO:0001658	branching involved in ureteric bud morphoproteinsis	1.72E-08	6.90E-07
GO:0009887	organ morphoproteinsis	2.72E-08	2.99E-14
GO:0001701	in utero embryonic development	2.89E-08	3.27E-14
GO:0030335	positive regulation of cell migration	4.24E-08	1.74E-05
GO:0030326	embryonic limb morphoproteinsis	6.92E-08	9.15E-40
GO:0045669	positive regulation of osteoblast differentiation	1.02E-07	8.74E-17
GO:0007507	heart development	2.36E-07	8.32E-18
GO:0060021	palate development	3.14E-07	8.09E-23
GO:0010718	positive regulation of epithelial to mesenchymal transition	3.16E-07	0.01455875
GO:0030509	BMP signaling pathway	3.53E-07	2.76E-12
GO:0045892	negative regulation of transcription, DNA-templated	3.57E-07	4.21E-07
GO:0042060	wound healing	5.21E-07	1.23E-05
GO:0048701	embryonic cranial skeleton morphoproteinsis	5.57E-07	8.42E-08
GO:0007275	multicellular organism development	5.67E-07	1.89E-21

GO:0001503	ossification	5.78E-07	1.14E-28
GO:0001934	positive regulation of protein phosphorylation	6.10E-07	1.08E-08
GO:0001501	skeletal system development	9.93E-07	4.60E-43
GO:0001657	ureteric bud development	1.30E-06	0.00213229
GO:0030324	lung development	2.06E-06	3.43E-11
GO:0000122	negative regulation of transcription from RNA polymerase II promoter	2.43E-06	7.84E-20
GO:0007389	pattern specification process	2.81E-06	4.16E-11
GO:0042475	odontoproteinsis of dentin-containing tooth	3.92E-06	6.87E-14
GO:0042733	embryonic digit morphoproteinsis	4.73E-06	5.30E-25
GO:0030500	regulation of bone mineralization	5.94E-06	1.79E-05
GO:0045165	cell fate commitment	6.70E-06	6.90E-07
GO:0060395	SMAD protein signal transduction	8.76E-06	9.49E-06
GO:0042474	middle ear morphoproteinsis	9.01E-06	0.00415561
GO:0051216	cartilage development	1.13E-05	6.37E-26
GO:0060070	canonical Wnt signaling pathway	1.56E-05	3.09E-09
GO:0001843	neural tube closure	2.19E-05	3.09E-06
GO:0090090	negative regulation of canonical Wnt signaling pathway	2.67E-05	4.37E-10
GO:0071773	cellular response to BMP stimulus	2.88E-05	0.00038339
GO:0001707	mesoderm formation	2.88E-05	4.37E-06
GO:0048754	branching morphoproteinsis of an epithelial tube	3.14E-05	5.48E-06
GO:0030501	positive regulation of bone mineralization	3.41E-05	3.34E-09
GO:0030879	mammary gland development	3.70E-05	7.76E-05
GO:0043066	negative regulation of apoptotic process	3.88E-05	3.04E-15
GO:0045596	negative regulation of cell differentiation	4.13E-05	0.01495266
GO:0030514	negative regulation of BMP signaling pathway	6.59E-05	0.03947855
GO:0048705	skeletal system morphoproteinsis	0.00010666	2.20E-09
GO:0043406	positive regulation of MAP kinase activity	0.00010666	0.00073993
GO:0045668	negative regulation of osteoblast differentiation	0.00011893	0.00015728
GO:0048762	mesenchymal cell differentiation	0.00015495	0.00017231
GO:0009612	response to mechanical stimulus	0.00016899	6.14E-05
GO:0061053	somite development	0.00025555	0.00048879
GO:0007179	transforming growth factor beta receptor signaling pathway	0.00028339	1.47E-07
GO:0070374	positive regulation of ERK1 and ERK2 cascade	0.00028588	3.19E-09
GO:0016477	cell migration	0.00030363	1.90E-06
GO:2000679	positive regulation of transcription regulatory region DNA binding	0.00033627	0.00934741
GO:0003203	endocardial cushion morphoproteinsis	0.0003807	0.00108701
GO:0016055	Wnt signaling pathway	0.00045893	1.58E-09

GO:0008283	cell proliferation	0.00051842	4.12E-06
GO:1902895	positive regulation of pri-miRNA transcription from RNA polymerase II promoter	0.00064319	0.02270901
GO:0032355	response to estradiol	0.00067863	4.88E-06
GO:0023019	signal transduction involved in regulation of gene expression	0.0007037	0.0035404
GO:0007267	cell-cell signaling	0.00071851	1.79E-07
GO:0031214	biomineral tissue development	0.00112204	0.00012938
GO:0001822	kidney development	0.00140877	2.39E-08
GO:0031016	pancreas development	0.00145299	2.71E-06
GO:0040007	growth	0.00172815	5.48E-06
GO:0002053	positive regulation of mesenchymal cell proliferation	0.00182495	2.88E-17
GO:0030154	cell differentiation	0.00184529	1.77E-08
GO:0007492	endoderm development	0.0021305	0.00497598
GO:0030901	midbrain development	0.0021305	1.26E-06
GO:0010595	positive regulation of endothelial cell migration	0.00223737	0.02729736
GO:0043065	positive regulation of apoptotic process	0.00245285	4.03E-10
GO:0045740	positive regulation of DNA replication	0.00245859	0.00679759
GO:0048706	embryonic skeletal system development	0.00257293	0.00020375
GO:0045597	positive regulation of cell differentiation	0.00280899	4.36E-06
GO:0010862	positive regulation of pathway-restricted SMAD protein phosphorylation	0.00305488	6.90E-07
GO:0045599	negative regulation of fat cell differentiation	0.00305488	6.90E-07
GO:0007568	aging	0.00317746	8.86E-08
GO:0048468	cell development	0.00318148	0.00040482
GO:0009953	dorsal/ventral pattern formation	0.00318148	4.20E-16
GO:0021915	neural tube development	0.00344197	1.25E-06
GO:0006355	regulation of transcription, DNA-templated	0.0035698	3.72E-08
GO:0001756	somitoproteinsis	0.00413529	3.60E-07
GO:0001666	response to hypoxia	0.00425666	8.11E-06
GO:0001541	ovarian follicle development	0.0042811	0.00102775
GO:0043408	regulation of MAPK cascade	0.00457982	0.00022813
GO:0071560	cellular response to transforming growth factor beta stimulus	0.00488793	4.73E-05
GO:0048812	neuron projection morphoproteinsis	0.00520538	0.00186241
GO:0003007	heart morphoproteinsis	0.00536758	1.28E-12
GO:0071363	cellular response to growth factor stimulus	0.00536758	6.98E-05
GO:0048839	inner ear development	0.0056989	8.92E-05
GO:0090263	positive regulation of canonical Wnt signaling pathway	0.00586801	3.47E-11
GO:0001938	positive regulation of endothelial cell proliferation	0.00586801	0.03967443

GO:0050680	negative regulation of epithelial cell proliferation	0.00674768	1.02E-11
GO:0000187	activation of MAPK activity	0.00749191	0.00128144
GO:0060548	negative regulation of cell death	0.00827159	0.02477654
GO:0048661	positive regulation of smooth muscle cell proliferation	0.00827159	1.38E-05
GO:0007050	cell cycle arrest	0.008472	1.56E-05
GO:0016485	protein processing	0.00950655	0.01045742
GO:0007417	central nervous system development	0.01015302	2.40E-08
GO:0061312	BMP signaling pathway involved in heart development	0.0102439	0.00041145
GO:0051897	positive regulation of protein kinase B signaling	0.01196962	0.00160387
GO:0050731	positive regulation of peptidyl-tyrosine phosphorylation	0.01292758	1.37E-07
GO:1904948	midbrain dopaminergic neuron differentiation	0.0136359	0.01964074
GO:0043410	positive regulation of MAPK cascade	0.01366739	7.62E-11
GO:0051092	positive regulation of NF-kappaB transcription factor activity	0.0149405	0.01163548
GO:0061384	heart trabecula morphoproteinsis	0.01532769	0.0247888
GO:0008584	male gonad development	0.0157281	7.20E-05
GO:0009952	anterior/posterior pattern specification	0.0157281	4.58E-17
GO:0006468	protein phosphorylation	0.01621133	3.88E-05
GO:0060039	pericardium development	0.01701666	0.03041854
GO:0007165	signal transduction	0.01815958	0.01179635
GO:0007435	salivary gland morphoproteinsis	0.01870283	0.00305644
GO:0007219	Notch signaling pathway	0.01934164	0.00078917
GO:0030308	negative regulation of cell growth	0.01934164	4.31E-07
GO:0042493	response to drug	0.01991886	3.17E-11
GO:0060272	embryonic skeletal joint morphoproteinsis	0.02038621	0.00025275
GO:0060389	pathway-restricted SMAD protein phosphorylation	0.02206678	0.0050812
GO:0006366	transcription from RNA polymerase II promoter	0.02326995	0.04072805
GO:0007411	axon guidance	0.02682883	9.88E-07
GO:0061036	positive regulation of cartilage development	0.02709179	0.00934741
GO:0035567	non-canonical Wnt signaling pathway	0.02876123	0.01111749
GO:0060038	cardiac muscle cell proliferation	0.02876123	0.01111749
GO:0035137	hindlimb morphoproteinsis	0.02876123	1.99E-07
GO:0060037	pharyngeal system development	0.0304279	0.01306762
GO:0017015	regulation of transforming growth factor beta receptor signaling pathway	0.03209181	0.00169332
GO:0046579	positive regulation of Ras protein signal transduction	0.03706695	0.02270901
GO:0006351	transcription, DNA-templated	0.03711583	4.28E-09

GO:0090190	positive regulation of branching involved in ureteric bud morphoproteinsis	0.03871983	3.17E-05
GO:0045778	positive regulation of ossification	0.03871983	5.75E-09
GO:0035050	embryonic heart tube development	0.03871983	0.02558431
GO:0009880	embryonic pattern specification	0.04036995	4.13E-05
GO:0048646	anatomical structure formation involved in morphoproteinsis	0.04036995	2.99E-06
GO:0045216	cell-cell junction organization	0.04036995	0.02864555
GO:0032967	positive regulation of collagen biosynthetic process	0.04366199	0.03532201
GO:0035987	endodermal cell differentiation	0.04530391	8.45E-05
GO:0042476	odontoproteinsis	0.04530391	5.02E-07
GO:0042981	regulation of apoptotic process	0.04548052	8.66E-07
GO:0035108	limb morphoproteinsis	0.0469431	1.11E-16
GO:0055010	ventricular cardiac muscle tissue morphoproteinsis	0.0469431	0.00097892
GO:0043392	negative regulation of DNA binding	0.04857957	0.00012938
GO:0071542	dopaminergic neuron differentiation	0.04857957	6.06E-08

Supplementary Table S11. The enriched Uberon terms that are common to the predicted proteins and proteins with original annotations for the pectoral fin. The enriched terms are sorted based on the p-value of those terms for the predicted proteins.

Uberon term identifier	Uberon term name	P-value for the predicted proteins	P-value for the original proteins
uberon_0011610	ceratohyal cartilage	3.81E-13	1.90E-14
uberon_0005886	post-hyoid pharyngeal arch skeleton	1.88E-12	2.56E-19
uberon_2001516	ceratobranchial cartilage	9.63E-11	0.00102618
uberon_0011242	ethmoid cartilage	9.63E-11	0.00029496
uberon_0003107	meckel's cartilage	1.84E-10	3.21E-12
uberon_0003079	floor plate	1.42E-09	0.03374939
uberon_0002533	post-anal tail bud	9.84E-08	0.00338073
uberon_0007215	trabecula cranii	1.50E-07	0.00035249
uberon_0008896	post-hyoid pharyngeal arch	1.85E-07	2.03E-11
uberon_0007812	post-anal tail	6.22E-07	0.00521186
uberon_0002329	somite	7.57E-07	0.00590808
uberon_0001016	nervous system	2.59E-06	0.03822373
uberon_0002328	notochord	6.81E-06	0.00808243
uberon_0011607	hyomandibular cartilage	1.03E-05	6.45E-06
uberon_0001049	neural tube	2.10E-05	0.0227542

uberon_0004752	palatoquadrate cartilage	2.14E-05	2.87E-09
uberon_2000250	opercle	2.74E-05	1.51E-05
uberon_0000165	mouth	3.80E-05	0.00014836
uberon_0000468	multicellular organism	4.20E-05	1.09E-09
uberon_0001894	diencephalon	5.15E-05	0.0201601
uberon_0003901	horizontal septum	7.47E-05	0.00192572
uberon_2001256	lateral floor plate	8.54E-05	0.03796748
uberon_0003051	ear vesicle	0.00019633	2.14E-06
uberon_0005945	neurocranial trabecula	0.0002281	0.00646992
uberon_2001239	ceratobranchial 5 bone	0.00033971	0.00024377
uberon_0003099	cranial neural crest	0.00040528	0.017947
uberon_0002028	hindbrain	0.0005451	0.00387881
uberon_0000044	dorsal root ganglion	0.00085431	0.03471745
uberon_0000965	lens of camera-type eye	0.00096984	0.02462029
uberon_0001890	forebrain	0.00100664	0.00629125
uberon_0001708	jaw skeleton	0.00115871	7.84E-20
uberon_2000694	ceratobranchial 5 tooth	0.00170211	7.74E-06
uberon_2001089	myoseptum	0.00170211	0.01399613
uberon_0002240	spinal cord	0.0017408	0.00254371
uberon_0001264	pancreas	0.00181488	0.00023088
uberon_0003936	postoptic commissure	0.00248307	0.00081772
uberon_0007329	pancreatic duct	0.003566	0.00556203
uberon_0011615	basihyal cartilage	0.00372936	0.0016559
uberon_0003077	paraxial mesoderm	0.00425656	0.00706401
uberon_2000040	median fin fold	0.0046312	1.80E-17
uberon_2000558	posterior macula	0.00500389	0.0087837
uberon_0000033	head	0.0061048	6.69E-08
uberon_0001032	sensory system	0.0062966	4.43E-05
uberon_0003072	optic cup	0.00666504	0.01290014
uberon_0003098	optic stalk	0.00666504	0.00167394
uberon_4000164	caudal fin	0.00707562	9.31E-11
uberon_2001069	ventral fin fold	0.00853811	2.60E-07
uberon_0009635	parachordal cartilage	0.00854196	3.23E-06
uberon_0004741	cleithrum	0.01174598	8.41E-06
uberon_0003278	skeleton of lower jaw	0.0124274	1.01E-06
uberon_0001898	hypothalamus	0.01291407	0.0053699
uberon_0003011	facial motor nucleus	0.01291407	0.0053699
uberon_0000926	mesoderm	0.01413075	0.03471745
uberon_0000948	heart	0.01508289	0.00333015

uberon_0001891	midbrain	0.01623867	0.01553909
uberon_0000019	camera-type eye	0.01756108	0.00298894
uberon_0005884	hyoid arch skeleton	0.0224617	0.00079602
uberon_0000935	anterior commissure	0.02393768	0.00050308
uberon_0011085	palatoquadrate arch	0.02551321	0.01749
uberon_0004375	bone of free limb or fin	0.03295212	0.0093632
uberon_0001703	neurocranium	0.03765523	1.19E-07
uberon_0001976	epithelium of esophagus	0.04102281	0.0137538
uberon_0002348	epicardium	0.04102281	0.00099481
uberon_2002193	dorsolateral septum	0.04102281	0.00099481
uberon_0011004	pharyngeal arch cartilage	0.04146017	2.83E-10
uberon_2001257	medial floor plate	0.04902763	0.0188574
uberon_0005598	trunk somite	0.04902763	0.0188574
uberon_0003114	pharyngeal arch 3	0.04902763	0.0188574

Supplementary Table S12. The enriched Uberon terms that are common to the predicted proteins and proteins with original annotations for the pelvic fin. The enriched terms are sorted based on the p-value of those terms for the predicted proteins.

Uberon term identifier	Uberon term name	P-value for the predicted proteins	P-value for the original proteins
uberon_0000151	pectoral fin	3.26E-07	1.42E-09
uberon_2000040	median fin fold	5.40E-06	8.70E-05
uberon_4000163	anal fin	0.00016979	4.43E-11
uberon_4000172	lepidotrichium	0.00351758	2.42E-06
uberon_0003097	dorsal fin	0.02306165	1.03E-11

Supplementary Table S13. The top 100 enriched Uberon terms that are common to the predicted proteins and proteins with original annotations for the forelimb. The enriched terms are sorted based on the p-value of those terms for the predicted proteins.

Uberon term identifier	Uberon term name	P-value for the predicted proteins	P-value for the original proteins
uberon_0001703	neurocranium	9.49E-19	3.67E-52

uberon_0011156	facial skeleton	2.21E-18	1.36E-65
uberon_0003128	cranium	5.88E-16	9.57E-79
uberon_0001676	occipital bone	7.46E-16	4.99E-20
uberon_0001708	jaw skeleton	2.83E-15	2.61E-61
uberon_0004716	conceptus	7.34E-14	2.32E-48
uberon_0007811	craniocervical region	7.49E-14	4.41E-77
uberon_0000209	tetrapod frontal bone	1.20E-13	1.61E-17
uberon_0002091	appendicular skeleton	1.27E-13	1.39E-187
uberon_0001434	skeletal system	2.87E-13	2.37E-122
uberon_0005944	axial skeleton plus cranial skeleton	3.25E-13	1.13E-87
uberon_0000165	mouth	1.11E-12	3.64E-61
uberon_0002517	basicranium	1.36E-12	2.14E-27
uberon_0001692	basioccipital bone	2.48E-12	0.00016976
uberon_0003252	thoracic rib cage	2.49E-12	4.72E-74
uberon_0001756	middle ear	3.11E-11	1.26E-24
uberon_0001456	face	5.09E-11	6.78E-61
uberon_0006428	basisphenoid bone	7.87E-11	5.22E-16
uberon_0001049	neural tube	1.06E-10	3.88E-29
uberon_0001684	mandible	1.12E-10	1.56E-51
uberon_0000210	tetrapod parietal bone	1.25E-10	1.03E-16
uberon_0003216	hard palate	1.35E-10	4.89E-27
uberon_0004747	supraoccipital bone	4.05E-10	6.38E-08
uberon_0000033	head	6.18E-10	2.24E-58
uberon_0001690	ear	6.79E-10	9.92E-30
uberon_0001716	secondary palate	1.22E-09	1.29E-40
uberon_0001685	hyoid bone	1.35E-09	1.21E-10
uberon_0001677	sphenoid bone	1.46E-09	8.95E-27
uberon_0001689	malleus bone	1.90E-09	8.78E-13
uberon_0002105	vestibulo-auditory system	2.07E-09	1.06E-28
uberon_0001007	digestive system	3.28E-09	1.27E-40
uberon_0002229	interparietal bone	4.28E-09	3.38E-18
uberon_0002228	rib	4.82E-09	2.08E-59
uberon_0001004	respiratory system	1.82E-08	9.08E-34
uberon_0000955	brain	2.00E-08	1.48E-14
uberon_0001678	temporal bone	2.39E-08	1.11E-14
uberon_0006721	alisphenoid bone	3.22E-08	5.08E-06
uberon_0003450	upper jaw incisor	6.27E-08	9.49E-07
uberon_0003051	ear vesicle	9.95E-08	1.97E-06
uberon_0008828	presphenoid bone	9.95E-08	1.88E-12

uberon_0000922	embryo	1.55E-07	1.90E-25
uberon_0003451	lower jaw incisor	1.66E-07	2.76E-08
uberon_0002218	tympanic ring	1.66E-07	1.04E-15
uberon_0002418	cartilage tissue	5.94E-07	8.02E-30
uberon_0003655	molar tooth	6.91E-07	1.66E-12
uberon_0003966	gonial bone	9.41E-07	0.00011778
uberon_0010389	pterygoid bone	9.41E-07	6.42E-06
uberon_0002510	anterior fontanel	1.34E-06	0.00254357
uberon_0002328	notochord	1.35E-06	2.01E-07
uberon_0001066	intervertebral disk	1.77E-06	2.31E-19
uberon_0011933	vibrissa unit	2.02E-06	3.00E-05
uberon_0001738	thyroid cartilage	2.79E-06	1.22E-07
uberon_0001681	nasal bone	3.03E-06	6.39E-22
uberon_0001890	forebrain	3.25E-06	3.76E-13
uberon_0004649	sphenoid bone pterygoid process	4.50E-06	1.93E-08
uberon_0002329	somite	4.85E-06	6.28E-09
uberon_0004660	mandible coronoid process	5.01E-06	4.14E-07
uberon_0005871	palatine process of maxilla	5.01E-06	1.37E-09
uberon_0001695	squamous part of temporal bone	6.79E-06	7.79E-07
uberon_0005942	hair outer root sheath	6.79E-06	0.01132058
uberon_0003075	neural plate	8.18E-06	0.00165765
uberon_0000924	ectoderm	8.52E-06	6.64E-17
uberon_0018242	palatine bone horizontal plate	9.74E-06	1.20E-07
uberon_0001694	petrous part of temporal bone	9.74E-06	2.04E-05
uberon_0002073	hair follicle	1.01E-05	7.57E-10
uberon_0002224	thoracic cavity	1.06E-05	9.88E-09
uberon_0000401	mandibular ramus	1.15E-05	4.55E-11
uberon_0001737	larynx	1.80E-05	1.91E-10
uberon_0006772	long bone epiphyseal plate hypertrophic zone	1.97E-05	2.91E-40
uberon_0001687	stapes bone	2.19E-05	6.84E-08
uberon_0003861	neural arch	2.26E-05	2.25E-18
uberon_0002470	autopod region	2.28E-05	5.54E-91
uberon_0002103	hindlimb	2.43E-05	2.57E-138
uberon_0001752	enamel	2.98E-05	0.00014902
uberon_0003107	meckel's cartilage	2.98E-05	4.98E-17
uberon_0002413	cervical vertebra	3.01E-05	1.16E-14
uberon_0001894	diencephalon	3.06E-05	5.38E-11
uberon_0001706	nasal septum	3.53E-05	1.69E-09
uberon_0001092	vertebral bone 1	3.73E-05	1.52E-10

uberon_0001682	palatine bone	3.93E-05	1.83E-10
uberon_0002416	integumental system	3.96E-05	2.80E-16
uberon_0000004	nose	4.01E-05	2.04E-24
uberon_0002028	hindbrain	4.59E-05	3.77E-08
uberon_0005354	malleus processus brevis	4.75E-05	0.00721838
uberon_0005619	secondary palatal shelf	4.82E-05	3.00E-17
uberon_0001130	vertebral column	5.03E-05	5.26E-27
uberon_0001691	external ear	5.83E-05	3.87E-15
uberon_0001232	collecting duct of renal tubule	6.68E-05	8.78E-05
uberon_0003982	mature ovarian follicle	6.68E-05	0.00364138
uberon_0001075	bony vertebral centrum	6.98E-05	6.89E-13
uberon_0001675	trigeminal ganglion	6.98E-05	0.000653
uberon_0001998	sternocostal joint	7.60E-05	1.87E-09
uberon_0003461	shoulder bone	8.04E-05	3.33E-19
uberon_0002129	cerebellar cortex	8.27E-05	0.00314678
uberon_0002414	lumbar vertebra	8.67E-05	6.55E-10
uberon_0005867	mandibular prominence	8.70E-05	0.00080115
uberon_0001037	strand of hair	0.00010094	3.10E-07
uberon_0003941	cerebellum anterior vermis	0.00010272	0.01504756
uberon_0002217	synovial joint	0.00010272	0.00100864
uberon_0001043	esophagus	0.00011262	6.33E-07

Supplementary Table S14. The top 100 enriched Uberon terms that are common to the predicted proteins and proteins with original annotations for the hindlimb. The enriched terms are sorted based on the p-value of those terms for the predicted proteins.

Uberon term identifier	Uberon term name	P-value for the predicted proteins	P-value for the original proteins
uberon_0003128	cranium	4.01E-24	5.82E-57
uberon_0011156	facial skeleton	1.11E-22	4.74E-53
uberon_0001434	skeletal system	1.42E-21	1.19E-223
uberon_0005944	axial skeleton plus cranial skeleton	4.39E-21	3.58E-88
uberon_0007811	craniocervical region	3.88E-19	2.99E-57
uberon_0001456	face	4.19E-19	2.62E-45
uberon_0000033	head	1.62E-18	5.98E-44
uberon_0001708	jaw skeleton	2.11E-17	1.39E-53
uberon_0001703	neurocranium	3.94E-17	2.49E-44

uberon_0000165	mouth	3.90E-16	1.59E-47
uberon_0002091	appendicular skeleton	1.44E-15	0
uberon_0002105	vestibulo-auditory system	1.25E-14	1.85E-25
uberon_0001690	ear	3.73E-14	1.27E-25
uberon_0001684	mandible	4.59E-14	8.20E-43
uberon_0001007	digestive system	1.41E-13	1.04E-28
uberon_0006333	snout	2.45E-13	8.50E-22
uberon_0002483	trabecular bone tissue	1.84E-12	4.59E-72
uberon_0000210	tetrapod parietal bone	1.91E-12	6.03E-14
uberon_0001756	middle ear	1.96E-12	9.35E-20
uberon_0001004	respiratory system	4.07E-12	8.92E-32
uberon_0000209	tetrapod frontal bone	3.47E-11	3.65E-13
uberon_0000383	musculature of body	4.96E-11	1.02E-56
uberon_0001630	muscle organ	1.56E-10	0.00011844
uberon_0002517	basicranium	3.84E-10	1.66E-17
uberon_0001049	neural tube	7.14E-10	2.88E-19
uberon_0001676	occipital bone	1.39E-09	1.36E-12
uberon_0002229	interparietal bone	1.95E-09	1.81E-10
uberon_0001677	sphenoid bone	2.11E-09	1.50E-13
uberon_0000955	brain	3.27E-09	2.55E-14
uberon_0006428	basisphenoid bone	4.44E-09	2.35E-07
uberon_0002228	rib	4.70E-09	4.14E-37
uberon_0003252	thoracic rib cage	9.23E-09	5.47E-50
uberon_0004716	conceptus	1.03E-08	3.81E-21
uberon_0003450	upper jaw incisor	1.09E-08	2.32E-05
uberon_0006772	long bone epiphyseal plate hypertrophic zone	2.06E-08	6.26E-55
uberon_0002416	integumental system	3.26E-08	9.53E-16
uberon_0003451	lower jaw incisor	3.59E-08	2.90E-09
uberon_0000014	zone of skin	4.54E-08	9.99E-19
uberon_0001689	malleus bone	5.05E-08	2.31E-12
uberon_0001130	vertebral column	5.84E-08	2.46E-37
uberon_0003107	meckel's cartilage	1.03E-07	2.75E-12
uberon_0004535	cardiovascular system	2.17E-07	5.30E-28
uberon_0002113	kidney	2.72E-07	3.61E-13
uberon_0006721	alisphenoid bone	4.21E-07	6.91E-06
uberon_0000474	female reproductive system	4.37E-07	7.29E-19
uberon_0003216	hard palate	4.55E-07	3.86E-17
uberon_0000019	camera-type eye	4.68E-07	1.67E-23
uberon_0002516	epiphyseal plate	4.92E-07	4.29E-72

uberon_0001678	temporal bone	6.21E-07	4.05E-12
uberon_0002418	cartilage tissue	6.60E-07	3.22E-14
uberon_0001890	forebrain	6.60E-07	2.57E-12
uberon_0005070	anterior neuropore	6.63E-07	0.04722767
uberon_0001692	basioccipital bone	9.19E-07	0.00016117
uberon_0002101	limb	9.91E-07	4.55E-08
uberon_0001008	renal system	1.01E-06	5.55E-21
uberon_0002104	visual system	1.18E-06	1.15E-25
uberon_0002397	maxilla	1.25E-06	8.65E-29
uberon_0001681	nasal bone	1.28E-06	2.59E-17
uberon_0002370	thymus	1.86E-06	5.49E-13
uberon_0002405	immune system	1.88E-06	1.05E-06
uberon_0003697	abdominal wall	2.07E-06	1.51E-07
uberon_0002218	tympanic ring	2.14E-06	5.31E-11
uberon_0001716	secondary palate	2.86E-06	2.15E-25
uberon_0000948	heart	2.92E-06	1.31E-18
uberon_0002390	hematopoietic system	3.26E-06	3.56E-07
uberon_0002544	digit	3.57E-06	2.75E-48
uberon_0003461	shoulder bone	4.45E-06	2.81E-14
uberon_0001752	enamel	5.00E-06	1.17E-06
uberon_0006849	scapula	5.00E-06	1.09E-18
uberon_0002491	lambdoid suture	5.12E-06	0.00065007
uberon_0003975	internal female genitalia	5.36E-06	5.15E-14
uberon_0000323	late embryo	8.00E-06	4.11E-10
uberon_0002428	limb bone	8.82E-06	0.00139297
uberon_0000309	body wall	9.97E-06	7.79E-08
uberon_0002048	lung	1.23E-05	3.26E-20
uberon_0002470	autopod region	1.37E-05	1.95E-55
uberon_0001229	renal corpuscle	1.41E-05	4.22E-05
uberon_0001075	bony vertebral centrum	1.53E-05	1.30E-09
uberon_0002328	notochord	1.71E-05	2.82E-07
uberon_0001285	nephron	1.79E-05	1.62E-06
uberon_0002073	hair follicle	1.82E-05	3.40E-07
uberon_0001225	cortex of kidney	2.68E-05	1.80E-05
uberon_0001987	placenta	2.68E-05	1.80E-05
uberon_0006861	diaphysis proper	3.28E-05	3.71E-12
uberon_0006771	long bone epiphyseal plate proliferative zone	3.45E-05	2.52E-29
uberon_0001016	nervous system	3.73E-05	5.57E-07
uberon_0005956	outflow part of left ventricle	3.78E-05	0.00017418

uberon_0001037	strand of hair	4.12E-05	3.95E-06
uberon_0002137	aortic valve	4.12E-05	0.00021436
uberon_0001695	squamous part of temporal bone	4.43E-05	0.00058499
uberon_0010166	coat of hair	5.19E-05	1.65E-06
uberon_0002329	somite	5.99E-05	0.00015746
uberon_0001758	periodontium	6.34E-05	3.57E-09
uberon_0003051	ear vesicle	6.89E-05	0.00027719
uberon_0008828	presphenoid bone	6.89E-05	3.51E-08
uberon_0001683	jugal bone	6.89E-05	8.05E-11
uberon_0002224	thoracic cavity	6.89E-05	5.32E-05
uberon_0002412	vertebra	7.08E-05	2.93E-18
uberon_0000945	stomach	7.42E-05	0.00021632
uberon_0000012	somatic nervous system	7.46E-05	1.69E-27

Supplementary Table S15. Comparison of the 37 proteins that are common to the pectoral fin and forelimb modules (conserved proteins). The proteins are ordered according to the rank in the pectoral fin module.

Zebrafish protein name	Annotation type	Weighted degree	Rank in the module	Mouse protein name	Annotation type	Weighted degree	Rank in the module
shha	direct annotation to the pectoral fin	36.6983	1	*shh*	direct annotation to the forelimb	37.6249	4
bmp4	predicted	35.5652	2	*bmp4*	direct annotation to the forelimb	47.2641	1
bmp2b	predicted	34.3109	3	*bmp2*	direct annotation to the forelimb	31.3004	13
wnt3a	predicted	31.4927	4	*wnt3a*	predicted	30.9124	14
fgf8a	predicted	31.0094	5	*fgf8*	direct annotation to the forelimb	34.0672	8
gli2a	predicted	27.2862	8	*gli2*	direct annotation to the forelimb	33.0563	11

bmp7a	direct annotation to the pectoral fin	25.8141	11	*bmp7*	predicted	34.2132	7
fgfr1a	predicted	25.6442	12	*fgfr1*	direct annotation to the forelimb	33.4360	10
fgf10a	direct annotation to the pectoral fin	24.2622	17	*fgf10*	direct annotation to the forelimb	22.7474	33
smo	direct annotation to the pectoral fin	23.6222	19	*smo*	direct annotation to the forelimb	28.7826	20
wnt4a	predicted	20.4183	29	*wnt4*	predicted	27.9668	23
ptch1	direct annotation to the pectoral fin	18.8304	30	*ptch1*	predicted	27.3374	24
ihha	predicted	18.1064	32	*ihh*	direct annotation to the forelimb	29.6653	17
tbx5a	direct annotation to the pectoral fin	16.7968	35	*tbx5*	direct annotation to the forelimb	8.6229	86
aldh1a2	direct annotation to the pectoral fin	16.6865	36	*aldh1a2*	annotated to a part or bud	7.1983	100
hand2	direct annotation to the pectoral fin	16.4984	38	*hand2*	direct annotation to the forelimb	13.8461	62
zic2a	predicted	15.2976	40	*zic2*	annotated to a part or bud	7.3701	99
tcf7	direct annotation to the pectoral fin	15.0392	42	*tcf7*	direct annotation to the forelimb	7.7495	93
msx1a	predicted	14.6308	45	*msx1*	predicted	23.4172	31
met	direct annotation to the pectoral fin	14.5234	46	*met*	direct annotation to the forelimb	13.9973	60
esr2a	predicted	13.2000	51	*esr2*	direct annotation to the forelimb	7.6748	95

apc	direct annotation to the pectoral fin	9.3808	61	*apc*	direct annotation to the forelimb	12.6243	67
tfap2a	predicted	9.2007	62	*tfap2a*	direct annotation to the forelimb	9.3891	78
dlx5a	annotated to a part or bud	8.9299	81	*dlx5*	predicted	16.1115	51
sox9a	direct annotation to the pectoral fin	8.8290	83	*sox9*	direct annotation to the forelimb	29.7370	15
disp1	direct annotation to the pectoral fin	8.6768	85	*disp1*	direct annotation to the forelimb	1.8115	183
mecom	predicted	8.4715	89	*mecom*	annotated to a part or bud	3.3452	155
sox9b	predicted	7.2558	104	*sox9*	direct annotation to the forelimb	29.7370	15
thraa	direct annotation to the pectoral fin	7.0911	105	*thra*	direct annotation to the forelimb	3.2473	157
cyp26b1	direct annotation to the pectoral fin	6.5056	111	*cyp26b1*	direct annotation to the forelimb	3.5223	151
ctgfa	direct annotation to the pectoral fin	5.3072	124	*ctgf*	direct annotation to the forelimb	20.3372	41
wls	direct annotation to the pectoral fin	4.8573	133	*wls*	direct annotation to the forelimb	6.6655	107
osr1	direct annotation to the pectoral fin	4.6429	136	*osr1*	annotated to a part or bud	0.3046	241
sparc	direct annotation to the pectoral fin	3.7395	148	*sparc*	direct annotation to the forelimb	12.4871	69
chsy1	direct annotation to the pectoral fin	1.9657	176	*chsy1*	annotated to a part or bud	1.0702	210

rspo2	direct annotation to the pectoral fin	0.7583	200	*rspo2*	direct annotation to the forelimb	3.6481	149
pax1b	direct annotation to the pectoral fin	0.3795	209	*pax1*	direct annotation to the forelimb	6.2887	111

Supplementary Table S16. Comparison of the 81 proteins that are common to the pelvic fin and hindlimb modules (conserved proteins). The proteins are ordered according to the rank in the pelvic fin module.

Zebrafish protein name	Annotation type	Weighted degree	Rank in the module	Mouse protein name	Annotation type	Weighted degree	Rank in the module
ctnnb1	predicted	106.2749	4	*ctnnb1*	direct annotation to the hindlimb	72.1808	3
smarca4a	predicted	97.4336	11	*smarca4*	direct annotation to the hindlimb	36.4034	56
kras	predicted	93.403	15	*kras*	predicted	70.7748	5
rac1a	predicted	92.9107	16	*rac1*	direct annotation to the hindlimb	24.2559	103
mapk14a	predicted	92.1789	17	*mapk14*	direct annotation to the hindlimb	71.7856	4
src	predicted	91.7256	18	*src*	direct annotation to the hindlimb	70.3811	6
fgfr1a	predicted	91.2553	19	*fgfr1*	direct annotation to the hindlimb	53.223	16
met	predicted	90.8735	20	*met*	direct annotation to the hindlimb	34.8637	64

tp53	predicted	83.8035	24	*trp53*	direct annotation to the hindlimb	82.9324	1
mtor	predicted	80.8121	28	*mtor*	annotated to a part or bud	38.6216	49
ptenb	predicted	78.531	32	*pten*	annotated to a part or bud	43.6208	35
bmp4	predicted	76.215	36	*bmp4*	direct annotation to the hindlimb	63.9989	10
bmp2b	predicted	68.2429	45	*bmp2*	predicted	45.0361	32
rac1b	predicted	67.0359	50	*rac1*	direct annotation to the hindlimb	24.2559	103
notch2	predicted	66.3735	54	*notch2*	direct annotation to the hindlimb	39.0854	47
fgfr2	predicted	63.0559	67	*fgfr2*	direct annotation to the hindlimb	48.7644	24
ptena	predicted	60.7765	80	*pten*	annotated to a part or bud	43.6208	35
mapk14b	predicted	59.24	89	*mapk14*	direct annotation to the hindlimb	71.7856	4
smad2	predicted	58.5213	92	*smad2*	predicted	45.6514	30
wnt3a	predicted	57.9542	95	*wnt3a*	direct annotation to the hindlimb	42.6564	39
ret	predicted	57.2523	98	*ret*	annotated to a part or bud	23.5326	110
vegfaa	predicted	57.0152	100	*vegfa*	annotated to a part or bud	50.7476	20
junba	predicted	54.8789	109	*junb*	direct annotation to the hindlimb	15.5932	205
fgf8a	predicted	52.5593	122	*fgf8*	direct annotation to the hindlimb	46.6654	29

gli2a	predicted	52.1448	130	*gli2*	direct annotation to the hindlimb	42.8396	38
dicer1	predicted	50.473	140	*dicer1*	direct annotation to the hindlimb	18.3287	187
smad3a	predicted	49.5403	150	*smad3*	annotated to a part or bud	49.4434	22
hrasb	predicted	48.0853	162	*hras*	predicted	72.8342	2
shha	predicted	48.0501	163	*shh*	direct annotation to the hindlimb	44.4006	33
bmp2a	predicted	47.8327	165	*bmp2*	predicted	45.0361	32
fgf4	predicted	47.5358	167	*fgf4*	annotated to a part or bud	24.3609	102
bmp7a	predicted	46.1288	178	*bmp7*	predicted	48.2316	26
vegfab	predicted	45.8605	183	*vegfa*	annotated to a part or bud	50.7476	20
mysm1	predicted	43.0713	209	*mysm1*	direct annotation to the hindlimb	4.6408	394
fgf10a	predicted	42.2153	212	*fgf10*	direct annotation to the hindlimb	33.825	67
fgf10b	predicted	40.7886	219	*fgf10*	direct annotation to the hindlimb	33.825	67
sirt1	predicted	40.1693	232	*sirt1*	annotated to a part or bud	25.4897	94
bmpr1aa	predicted	36.7892	251	*bmpr1a*	direct annotation to the hindlimb	35.5242	61
lef1	predicted	35.665	259	*lef1*	predicted	36.7074	53
gli2b	predicted	33.6578	277	*gli2*	direct annotation to the hindlimb	42.8396	38
wnt4a	predicted	33.0982	286	*wnt4*	predicted	35.4014	62
smo	predicted	32.2549	293	*smo*	direct annotation to the hindlimb	34.086	66

sod2	predicted	30.6061	308	*sod2*	annotated to a part or bud	18.8196	178
gdf11	direct annotation to the pelvic fin	29.3173	321	*gdf11*	direct annotation to the hindlimb	8.6569	283
stat1b	predicted	28.9557	323	*stat1*	direct annotation to the hindlimb	33.3038	72
smad3b	predicted	27.6166	333	*smad3*	annotated to a part or bud	49.4434	22
casp3a	predicted	27.2208	340	*casp3*	predicted	49.0774	23
map3k14b	predicted	26.1706	350	*map3k14*	annotated to a part or bud	7.1186	312
aldh1a2	predicted	25.4504	354	*aldh1a2*	annotated to a part or bud	9.8933	263
gli3	predicted	25.0939	359	*gli3*	direct annotation to the hindlimb	38.3084	52
bmpr1ba	predicted	24.8781	361	*bmpr1b*	annotated to a part or bud	20.0326	131
ihha	predicted	24.0223	366	*ihh*	direct annotation to the hindlimb	33.7444	68
ptch1	predicted	23.8507	369	*ptch1*	predicted	38.8191	48
bmpr1ab	predicted	22.5899	386	*bmpr1a*	direct annotation to the hindlimb	35.5242	61
vdrb	predicted	22.4408	388	*vdr*	direct annotation to the hindlimb	15.8149	202
shhb	predicted	20.6403	404	*shh*	direct annotation to the hindlimb	44.4006	33
bmpr1bb	predicted	20.1116	408	*bmpr1b*	annotated to a part or bud	20.0326	131
fsta	predicted	17.8232	422	*fst*	annotated to a part or bud	27.0851	86
hand2	predicted	15.7932	437	*hand2*	direct annotation	24.7719	101

					to the hindlimb		
msx1a	predicted	15.3589	442	*msx1*	predicted	26.6468	88
cx43	predicted	15.0787	444	*gja1*	predicted	28.3709	84
vdra	predicted	13.7382	454	*vdr*	direct annotation to the hindlimb	15.8149	202
pax3a	predicted	13.4122	458	*pax3*	direct annotation to the hindlimb	31.1909	76
ihhb	predicted	13.389	460	*ihh*	direct annotation to the hindlimb	33.7444	68
lamc1	predicted	13.3297	461	*lamc1*	direct annotation to the hindlimb	7.8573	299
desma	predicted	13.1643	462	*des*	annotated to a part or bud	14.3164	217
col1a1a	predicted	12.9905	465	*col1a1*	direct annotation to the hindlimb	43.3734	36
lmx1bb	predicted	11.9354	481	*lmx1b*	annotated to a part or bud	9.1733	271
col2a1a	predicted	11.8949	483	*col2a1*	direct annotation to the hindlimb	43.3479	37
tbx4	direct annotation to the pelvic fin	10.3408	496	*tbx4*	direct annotation to the hindlimb	10.3871	259
hspg2	predicted	9.6841	500	*hspg2*	direct annotation to the hindlimb	14.1006	220
fras1	predicted	9.0362	507	*fras1*	annotated to a part or bud	14.535	212
col1a2	predicted	6.6976	539	*col1a2*	direct annotation to the hindlimb	28.4794	83
scube2	predicted	6.4974	543	*scube2*	direct annotation to the hindlimb	3.4888	434

col11a1b	predicted	6.2965	545	*col11a1*	direct annotation to the hindlimb	14.3918	215
col11a1a	predicted	5.9988	550	*col11a1*	direct annotation to the hindlimb	14.3918	215
col1a1b	predicted	5.9171	554	*col1a1*	direct annotation to the hindlimb	43.3734	36
rspo2	annotated to a part or bud	4.0927	570	*rspo2*	direct annotation to the hindlimb	3.6481	429
frem2a	predicted	3.5366	577	*frem2*	annotated to a part or bud	1.5047	537
pitx1	predicted	2.8035	592	*pitx1*	direct annotation to the hindlimb	9.5149	267
pax3b	predicted	2.4374	595	*pax3*	direct annotation to the hindlimb	31.1909	76

Supplementary Table S17. The enriched Biological Process terms from the Gene Ontology for the mouse orthologs of the pectoral fin module-specific proteins.

Term identifier	Term name	P-value
GO:0007275	multicellular organism development	5.68E-14
GO:0045944	positive regulation of transcription from RNA polymerase II promoter	3.32E-10
GO:0009887	organ morphoproteinsis	6.91E-09
GO:0006355	regulation of transcription, DNA-templated	4.00E-08
GO:0006351	transcription, DNA-templated	3.72E-07
GO:0060070	canonical Wnt signaling pathway	4.94E-07
GO:0043588	skin development	6.59E-07
GO:0042475	odontoproteinsis of dentin-containing tooth	1.08E-06
GO:0006357	regulation of transcription from RNA polymerase II promoter	1.41E-06
GO:0045893	positive regulation of transcription, DNA-templated	1.22E-05
GO:0030154	cell differentiation	1.67E-05
GO:0016055	Wnt signaling pathway	1.91E-05

GO:0021766	hippocampus development	4.51E-05
GO:0010628	positive regulation of gene expression	5.57E-05
GO:0000122	negative regulation of transcription from RNA polymerase II promoter	1.37E-04
GO:0090090	negative regulation of canonical Wnt signaling pathway	2.21E-04
GO:0035904	aorta development	3.56E-04
GO:0006024	glycosaminoglycan biosynthetic process	4.45E-04
GO:0021522	spinal cord motor neuron differentiation	4.94E-04
GO:0007507	heart development	5.13E-04
GO:0007224	smoothened signaling pathway	5.74E-04
GO:0030182	neuron differentiation	6.07E-04
GO:0043010	camera-type eye development	8.19E-04
GO:0060021	palate development	0.00113011
GO:0007281	germ cell development	0.00128033
GO:0001843	neural tube closure	0.00184155
GO:0002062	chondrocyte differentiation	0.00192196
GO:0001657	ureteric bud development	0.00217299
GO:0008284	positive regulation of cell proliferation	0.00252077
GO:0001756	somitoproteinsis	0.00337659
GO:0030111	regulation of Wnt signaling pathway	0.00412055
GO:0015012	heparan sulfate proteoglycan biosynthetic process	0.00412055
GO:0030324	lung development	0.00446791
GO:0071300	cellular response to retinoic acid	0.00513915
GO:0001701	in utero embryonic development	0.00546615
GO:0045669	positive regulation of osteoblast differentiation	0.00582864
GO:0001568	blood vessel development	0.00657098
GO:0045165	cell fate commitment	0.00683035
GO:0048844	artery morphoproteinsis	0.00788494
GO:0045665	negative regulation of neuron differentiation	0.00792833
GO:0030334	regulation of cell migration	0.00821812
GO:0051216	cartilage development	0.00976019
GO:0043066	negative regulation of apoptotic process	0.01109612
GO:0021983	pituitary gland development	0.0112363
GO:0001837	epithelial to mesenchymal transition	0.01196962
GO:0007417	central nervous system development	0.01218445
GO:0045892	negative regulation of transcription, DNA-templated	0.01259202
GO:0035116	embryonic hindlimb morphoproteinsis	0.01349739
GO:0010718	positive regulation of epithelial to mesenchymal transition	0.01429137
GO:0007399	nervous system development	0.01594534
GO:0061153	trachea gland development	0.01600806

GO:0060976	coronary vasculature development	0.01679091
GO:0001656	metanephros development	0.01766254
GO:0003281	ventricular septum development	0.01766254
GO:0071787	endoplasmic reticulum tubular network assembly	0.02128751
GO:0034653	retinoic acid catabolic process	0.02128751
GO:0008543	fibroblast growth factor receptor signaling pathway	0.02229888
GO:0001649	osteoblast differentiation	0.02292059
GO:0048706	embryonic skeletal system development	0.02328026
GO:0002051	osteoblast fate commitment	0.02653893
GO:0048755	branching morphoproteinsis of a nerve	0.02653893
GO:0001755	neural crest cell migration	0.02844596
GO:0001942	hair follicle development	0.03174493
GO:0072177	mesonephric duct development	0.03176245
GO:2000343	positive regulation of chemokine (C-X-C motif) ligand 2 production	0.03176245
GO:0060662	salivary gland cavitation	0.03176245
GO:0060789	hair follicle placode formation	0.03176245
GO:0042127	regulation of cell proliferation	0.0339043
GO:0060348	bone development	0.03636502
GO:0009913	epidermal cell differentiation	0.03695824
GO:1901522	positive regulation of transcription from RNA polymerase II promoter involved in cellular response to chemical stimulus	0.03695824
GO:0021554	optic nerve development	0.03695824
GO:0071168	protein localization to chromatin	0.03695824
GO:0048752	semicircular canal morphoproteinsis	0.03695824
GO:0048793	pronephros development	0.03695824
GO:0048341	paraxial mesoderm formation	0.04212643
GO:0048702	embryonic neurocranium morphoproteinsis	0.04212643
GO:0030853	negative regulation of granulocyte differentiation	0.04212643
GO:0001947	heart looping	0.04374438
GO:0016567	protein ubiquitination	0.04533511

Supplementary Table S18. The top 100 enriched Uberon terms for the mouse orthologs of the pectoral fin module-specific proteins.

Uberon identifier	Term name	P-value
uberon 0000922	embryo	6.92E-12
uberon 0004716	conceptus	2.01E-11
uberon 0007811	craniocervical region	8.10E-11

uberon 0001049	neural tube	3.87E-10
uberon 0005944	axial skeleton plus cranial skeleton	1.06E-09
uberon 0003128	cranium	1.35E-09
uberon 0000033	head	1.99E-09
uberon 0001456	face	5.43E-08
uberon 0001434	skeletal system	5.81E-07
uberon 0000165	mouth	5.91E-07
uberon 0001703	neurocranium	1.11E-06
uberon 0001007	digestive system	2.10E-06
uberon 0000926	mesoderm	2.55E-06
uberon 0001711	eyelid	2.75E-06
uberon 0004341	primitive streak	3.74E-06
uberon 0011156	facial skeleton	5.55E-06
uberon 0003457	head bone	7.51E-06
uberon 0005070	anterior neuropore	1.28E-05
uberon 0001819	palpebral fissure	2.93E-05
uberon 0001708	jaw skeleton	2.93E-05
uberon 0000478	extraembryonic structure	3.52E-05
uberon 0000947	aorta	4.31E-05
uberon 0003655	molar tooth	5.25E-05
uberon 0004022	germinal neuroepithelium	7.58E-05
uberon 0000948	heart	9.02E-05
uberon 0000019	camera-type eye	9.15E-05
uberon 0002104	visual system	9.22E-05
uberon 0005600	crus commune	9.42E-05
uberon 0001860	endolymphatic duct	0.0001457
uberon 0003451	lower jaw incisor	0.00017578
uberon 0002470	autopod region	0.00019574
uberon 0001508	arch of aorta	0.0002267
uberon 0002167	right lung	0.00023623
uberon 0001004	respiratory system	0.0002535
uberon 0001690	ear	0.00026285
uberon 0002105	vestibulo-auditory system	0.00028019
uberon 0003216	hard palate	0.00029064
uberon 0010190	pair of dorsal aortae	0.00029282
uberon 0002384	connective tissue	0.00030192
uberon 0004044	anterior visceral endoderm	0.00043905
uberon 0001818	tarsal gland	0.00049939
uberon 0035077	lateral nasal gland	0.00062633

uberon 0000014	zone of skin	0.0006311
uberon 0002544	digit	0.00066645
uberon 0002827	vestibulocochlear ganglion	0.00071149
uberon 0003955	molar crown	0.0007931
uberon 0001756	middle ear	0.00080729
uberon 0004535	cardiovascular system	0.00087226
uberon 0000423	eccrine sweat gland	0.00087311
uberon 0001601	extra-ocular muscle	0.00087311
uberon 0000004	nose	0.00090415
uberon 0002539	pharyngeal arch	0.00091495
uberon 0000084	ureteric bud	0.0009682
uberon 0001751	dentine	0.00097362
uberon 0003066	pharyngeal arch 2	0.00097362
uberon 0002168	left lung	0.00120309
uberon 0001688	incus bone	0.00128991
uberon 0000091	bilaminar disc	0.00140356
uberon 0011864	tendon collagen fibril	0.00148402
uberon 0001716	secondary palate	0.00151298
uberon 0003544	brain white matter	0.0016391
uberon 0000955	brain	0.00172361
uberon 0003051	ear vesicle	0.00180251
uberon 0003068	axial mesoderm	0.00184711
uberon 0007833	osseus semicircular canal	0.00187241
uberon 0000309	body wall	0.00193054
uberon 0002091	appendicular skeleton	0.00207267
uberon 0004573	systemic artery	0.00222527
uberon 0010513	strand of zigzag hair	0.00225969
uberon 0000945	stomach	0.0023366
uberon 0010409	ocular surface region	0.00233819
uberon 0004043	semicircular canal ampulla	0.00242659
uberon 0005356	rathke's pouch	0.00242659
uberon 0003950	inner ear canal	0.00254497
uberon 0001890	forebrain	0.00257106
uberon 0003252	thoracic rib cage	0.00267041
uberon 0008854	root of molar tooth	0.00268605
uberon 0008799	transverse palatine fold	0.00268605
uberon 0002418	cartilage tissue	0.00271496
uberon 0001199	mucosa of stomach	0.00287821
uberon 0005291	embryonic tissue	0.00300689

uberon 0001167	wall of stomach	0.00324016
uberon 0000007	pituitary gland	0.00332109
uberon 0001862	vestibular labyrinth	0.003632
uberon 0002413	cervical vertebra	0.00380992
uberon 0004362	pharyngeal arch 1	0.00403845
uberon 0001872	parietal lobe	0.00403845
uberon 0002329	somite	0.00405486
uberon 0001908	optic tract	0.00421892
uberon 0001274	ischium	0.00421892
uberon 0001853	utricle of membranous labyrinth	0.00477616
uberon 0001752	enamel	0.00477616
uberon 0002196	adenohypophysis	0.00516843
uberon 0005176	tooth enamel organ	0.00541814
uberon 0002487	tooth cavity	0.00541814
uberon 0010197	trunk of common carotid artery	0.00541814
uberon 0004090	periorbital region	0.00541814
uberon 0001894	diencephalon	0.00586178
uberon 0001854	saccule of membranous labyrinth	0.00617638
uberon 0004212	glomerular capillary	0.00617638

Supplementary Table S19. The top 100 enriched Biological Process terms from the Gene Ontology for the mouse orthologs of the pelvic fin module-specific proteins.

Term identifier	Term name	P-value
GO:0006468	protein phosphorylation	5.19E-34
GO:0016310	phosphorylation	1.24E-30
GO:0046777	protein autophosphorylation	9.64E-22
GO:0018105	peptidyl-serine phosphorylation	1.63E-15
GO:0045893	positive regulation of transcription, DNA-templated	5.38E-15
GO:0018108	peptidyl-tyrosine phosphorylation	1.80E-14
GO:0045944	positive regulation of transcription from RNA polymerase II promoter	3.24E-14
GO:0007169	transmembrane receptor protein tyrosine kinase signaling pathway	1.41E-13
GO:0010628	positive regulation of gene expression	1.67E-13
GO:0009887	organ morphoproteinsis	7.65E-12
GO:0030335	positive regulation of cell migration	1.14E-11
GO:0042493	response to drug	7.06E-11
GO:0018107	peptidyl-threonine phosphorylation	1.51E-10
GO:0043066	negative regulation of apoptotic process	4.82E-10

GO:0043627	response to estrogen	5.96E-10
GO:0001934	positive regulation of protein phosphorylation	6.88E-10
GO:0006351	transcription, DNA-templated	1.09E-09
GO:0038083	peptidyl-tyrosine autophosphorylation	1.75E-09
GO:0048565	digestive tract development	2.26E-09
GO:0035690	cellular response to drug	7.15E-09
GO:0008284	positive regulation of cell proliferation	9.34E-09
GO:0035556	intracellular signal transduction	1.19E-08
GO:0030154	cell differentiation	1.47E-08
GO:0035970	peptidyl-threonine dephosphorylation	2.43E-08
GO:0007275	multicellular organism development	4.69E-08
GO:0045665	negative regulation of neuron differentiation	8.95E-08
GO:0071773	cellular response to BMP stimulus	1.34E-07
GO:0007264	small GTPase mediated signal transduction	2.17E-07
GO:0000122	negative regulation of transcription from RNA polymerase II promoter	2.52E-07
GO:0030182	neuron differentiation	3.94E-07
GO:0060070	canonical Wnt signaling pathway	4.66E-07
GO:0071407	cellular response to organic cyclic compound	7.62E-07
GO:0016477	cell migration	1.56E-06
GO:0006355	regulation of transcription, DNA-templated	1.98E-06
GO:0007178	transmembrane receptor protein serine/threonine kinase signaling pathway	2.25E-06
GO:0001942	hair follicle development	3.29E-06
GO:0043525	positive regulation of neuron apoptotic process	3.31E-06
GO:0007049	cell cycle	4.64E-06
GO:0043524	negative regulation of neuron apoptotic process	5.40E-06
GO:0007179	transforming growth factor beta receptor signaling pathway	6.68E-06
GO:0006357	regulation of transcription from RNA polymerase II promoter	8.40E-06
GO:0048485	sympathetic nervous system development	8.61E-06
GO:0045740	positive regulation of DNA replication	9.30E-06
GO:0071363	cellular response to growth factor stimulus	1.61E-05
GO:0007507	heart development	1.70E-05
GO:0048663	neuron fate commitment	1.77E-05
GO:0030509	BMP signaling pathway	2.27E-05
GO:0001701	in utero embryonic development	2.45E-05
GO:0008584	male gonad development	2.93E-05
GO:0006470	protein dephosphorylation	3.41E-05
GO:0071333	cellular response to glucose stimulus	3.48E-05
GO:0030513	positive regulation of BMP signaling pathway	3.59E-05
GO:0031175	neuron projection development	3.65E-05

GO:0045165	cell fate commitment	3.86E-05
GO:0003151	outflow tract morphoproteinsis	4.33E-05
GO:0043406	positive regulation of MAP kinase activity	4.33E-05
GO:0042127	regulation of cell proliferation	5.34E-05
GO:0010629	negative regulation of gene expression	7.71E-05
GO:0016569	covalent chromatin modification	8.05E-05
GO:0051496	positive regulation of stress fiber assembly	1.15E-04
GO:0060041	retina development in camera-type eye	1.17E-04
GO:0010468	regulation of gene expression	1.23E-04
GO:0003007	heart morphoproteinsis	1.31E-04
GO:0045429	positive regulation of nitric oxide biosynthetic process	1.31E-04
GO:0007399	nervous system development	1.38E-04
GO:0045597	positive regulation of cell differentiation	1.49E-04
GO:0006366	transcription from RNA polymerase II promoter	1.71E-04
GO:0008285	negative regulation of cell proliferation	1.73E-04
GO:0007165	signal transduction	2.05E-04
GO:0045931	positive regulation of mitotic cell cycle	2.26E-04
GO:0032212	positive regulation of telomere maintenance via telomerase	2.26E-04
GO:0045471	response to ethanol	2.30E-04
GO:0007224	smoothened signaling pathway	2.52E-04
GO:0007167	enzyme linked receptor protein signaling pathway	2.85E-04
GO:0007204	positive regulation of cytosolic calcium ion concentration	3.02E-04
GO:0048709	oligodendrocyte differentiation	3.03E-04
GO:0001525	angioproteinsis	3.37E-04
GO:0030324	lung development	3.55E-04
GO:0001764	neuron migration	3.55E-04
GO:0048015	phosphatidylinositol-mediated signaling	3.99E-04
GO:0048538	thymus development	4.03E-04
GO:0016055	Wnt signaling pathway	4.15E-04
GO:0090090	negative regulation of canonical Wnt signaling pathway	4.48E-04
GO:0010001	glial cell differentiation	4.49E-04
GO:0071260	cellular response to mechanical stimulus	4.50E-04
GO:0030501	positive regulation of bone mineralization	4.54E-04
GO:0009790	embryo development	4.87E-04
GO:0070301	cellular response to hydrogen peroxide	4.90E-04
GO:0045909	positive regulation of vasodilation	5.16E-04
GO:0060412	ventricular septum morphoproteinsis	5.16E-04
GO:0070374	positive regulation of ERK1 and ERK2 cascade	5.21E-04
GO:0048661	positive regulation of smooth muscle cell proliferation	5.26E-04

GO:0008283	cell proliferation	5.53E-04
GO:0051091	positive regulation of sequence-specific DNA binding transcription factor activity	6.17E-04
GO:0001666	response to hypoxia	6.21E-04
GO:0060045	positive regulation of cardiac muscle cell proliferation	6.46E-04
GO:0007623	circadian rhythm	6.56E-04
GO:0048646	anatomical structure formation involved in morphoproteinsis	7.64E-04
GO:0048854	brain morphoproteinsis	7.64E-04
GO:0000082	G1/S transition of mitotic cell cycle	7.71E-04

Supplementary Table S20. The top 100 Uberon terms for the mouse orthologs of the pelvic fin module-specific proteins.

Uberon identifier	Term name	P-value
uberon 0000922	embryo	6.50E-16
uberon 0004716	conceptus	1.53E-13
uberon 0004365	vitelline blood vessel	2.69E-09
uberon 0000478	extraembryonic structure	3.51E-09
uberon 0001007	digestive system	8.53E-09
uberon 0000014	zone of skin	2.47E-08
uberon 0010190	pair of dorsal aortae	4.77E-08
uberon 0001049	neural tube	1.02E-07
uberon 0008852	visceral yolk sac	1.23E-07
uberon 0000948	heart	1.60E-07
uberon 0004535	cardiovascular system	4.34E-07
uberon 0000468	multicellular organism	5.93E-07
uberon 0000016	endocrine pancreas	8.87E-07
uberon 0002067	dermis	1.91E-06
uberon 0001004	respiratory system	3.10E-06
uberon 0000358	blastocyst	3.19E-06
uberon 0000033	head	3.57E-06
uberon 0002012	pulmonary artery	4.49E-06
uberon 0002407	pericardium	1.34E-05
uberon 0002048	lung	1.66E-05
uberon 0004374	vitelline vasculature	2.27E-05
uberon 0001987	placenta	2.69E-05
uberon 0003087	anterior cardinal vein	3.00E-05

uberon 0000006	islet of langerhans	3.49E-05
uberon 0001264	pancreas	4.05E-05
uberon 0000947	aorta	4.50E-05
uberon 0001456	face	6.01E-05
uberon 0003512	lung blood vessel	6.31E-05
uberon 0002240	spinal cord	7.35E-05
uberon 0003946	placenta labyrinth	0.00010402
uberon 0002368	endocrine gland	0.00011026
uberon 0001792	ganglionic layer of retina	0.00012935
uberon 0002062	endocardial cushion	0.00014493
uberon 0001508	arch of aorta	0.00015353
uberon 0001083	myocardium of ventricle	0.0001601
uberon 0007811	craniocervical region	0.00016979
uberon 0001809	enteric ganglion	0.0001807
uberon 0000084	ureteric bud	0.00020974
uberon 0002087	atrioventricular canal	0.00022946
uberon 0002315	gray matter of spinal cord	0.00024038
uberon 0004647	liver lobule	0.00026764
uberon 0001818	tarsal gland	0.00026935
uberon 0000165	mouth	0.00027721
uberon 0001675	trigeminal ganglion	0.00027999
uberon 0006207	aortico-pulmonary spiral septum	0.00028524
uberon 0002416	integumental system	0.00028781
uberon 0010172	bulb of aorta	0.00030071
uberon 0002005	enteric nervous system	0.00030515
uberon 0006524	alveolar system	0.00031454
uberon 0008870	pulmonary alveolar parenchyma	0.00034866
uberon 0000088	trophoblast	0.00039593
uberon 0002094	interventricular septum	0.000402
uberon 0000383	musculature of body	0.00042772
uberon 0001711	eyelid	0.00043278
uberon 0003216	hard palate	0.00044958
uberon 0001280	liver parenchyma	0.00046467
uberon 0000011	parasympathetic nervous system	0.0004649
uberon 0002073	hair follicle	0.00052472
uberon 0002370	thymus	0.00057905
uberon 0008856	stomach muscularis externa	0.0006099
uberon 0000019	camera-type eye	0.00061682
uberon 0002408	parietal serous pericardium	0.00062547

uberon 0001496	ascending aorta	0.00063348
uberon 0000117	respiratory tube	0.00063848
uberon 0002165	endocardium	0.00073687
uberon 0003066	pharyngeal arch 2	0.00073687
uberon 0002511	trabecula carnea	0.00077776
uberon 0000013	sympathetic nervous system	0.0008281
uberon 0000012	somatic nervous system	0.00090485
uberon 0002342	neural crest	0.00093289
uberon 0003618	aorta tunica media	0.00093289
uberon 0002080	heart right ventricle	0.00100621
uberon 0001637	artery	0.0010228
uberon 0001708	jaw skeleton	0.00113097
uberon 0002104	visual system	0.00120939
uberon 0002069	stratum granulosum of epidermis	0.00121228
uberon 0001074	pericardial cavity	0.00142689
uberon 0001041	foregut	0.00145277
uberon 0008874	pulmonary acinus	0.00148651
uberon 0004493	cardiac muscle tissue of myocardium	0.00151061
uberon 0002410	autonomic nervous system	0.00154176
uberon 0001135	smooth muscle tissue	0.00168825
uberon 0001716	secondary palate	0.00180283
uberon 0003501	retina blood vessel	0.00195196
uberon 0002025	stratum basale of epidermis	0.00203946
uberon 0011156	facial skeleton	0.0021246
uberon 0003823	hindlimb zeugopod	0.00219172
uberon 0001534	right subclavian artery	0.00222747
uberon 0005970	brain commissure	0.00223824
uberon 0003073	lens placode	0.00223824
uberon 0003128	cranium	0.00240653
uberon 0001003	skin epidermis	0.0024443
uberon 0010513	strand of zigzag hair	0.00258297
uberon 0001213	intestinal villus	0.00269833
uberon 0001806	sympathetic ganglion	0.00286072
uberon 0005870	olfactory pit	0.00286072
uberon 0005343	cortical plate	0.00286898
uberon 0005062	neural fold	0.00286898
uberon 0004663	aorta wall	0.0028703
uberon 0010512	strand of guard hair	0.00311381

Supplementary Table S21. Some of the enriched GO-BP and Uberon terms for the mouse orthologs of the pectoral fin module-specific proteins that are related to novel anatomical entities emerged in tetrapods during fin to limb transition. The terms are organized into specific anatomical regions.

Anatomical region	Term Identifier	Term name	P-value
Lung	uberon 0002167	right lung	0.00023623
	uberon 0002168	left lung	0.00120309
	uberon 0003512	lung blood vessel	0.04982657
	GO:0030324	lung development	0.00446791
Neck and lower jaw	uberon 0001708	jaw skeleton	2.93E-05
	uberon 0003451	lower jaw incisor	0.00017578
	uberon 0002413	cervical vertebra	0.00380992
	uberon 0003216	hard palate	0.00029064
	uberon 0001716	secondary palate	0.00151298
	GO:0060021	palate development	0.00113011
	GO:0061153	trachea gland development	0.01600806
Face and hair	uberon 0001456	face	5.43E-08
	uberon 0005600	crus commune	9.42E-05
	uberon 0001690	ear	0.00026285
	uberon 0000004	nose	0.00090415
	uberon 0001711	eyelid	2.75E-06
	uberon 0034772	margin of eyelid	0.03232832
	uberon 0002073	hair follicle	0.02808325
	uberon 0010514	strand of duvet hair	0.02594653
	GO:0001942	hair follicle development	0.03174493
	GO:0060789	hair follicle placode formation	0.03176245
	GO:0061029	eyelid development in camera-type eye	0.07754317
Other	uberon 0002544	digit	0.00066645

Supplementary Table S22. Some of the enriched GO-BP and Uberon terms for the mouse orthologs of the pelvic fin module-specific proteins that are related to novel anatomical entities emerged in tetrapods during fin to limb transition. The terms are organized into specific anatomical regions.

Anatomical region	Term Identifier	Term name	P-value
Lung	uberon 0001004	respiratory system	3.10E-06
	uberon 0002012	pulmonary artery	4.49E-06
	uberon 0002048	lung	1.66E-05
	uberon 0003512	lung blood vessel	6.31E-05
	uberon 0006524	alveolar system	0.00031454
	uberon 0008870	pulmonary alveolar parenchyma	0.00034866
	uberon 0000117	respiratory tube	0.00063848
	uberon 0004785	respiratory system mucosa	0.00465107
	GO:0030324	lung development	3.55E-04
	GO:0060425	lung morphoproteinsis	0.0083659
Neck and lower jaw	uberon 0003216	hard palate	0.00044958
	uberon 0002370	thymus	0.00057905
	GO:0048538	thymus development	4.03E-04
	GO:0060021	palate development	0.00391519
	GO:0060440	trachea formation	0.00614484
	GO:0060017	parathyroid gland development	0.00614484
Face and hair	uberon 0001818	tarsal gland	0.00026935
	uberon 0001711	eyelid	0.00043278
	uberon 0000004	nose	0.00707171
	uberon 0001681	nasal bone	0.01712347
	uberon 0002073	hair follicle	0.00052472
	uberon 0010512	strand of guard hair	0.00311381
	GO:0061029	eyelid development in camera-type eye	0.00211434
	GO:0001942	hair follicle development	3.29E-06
Other	uberon 0001987	placenta	2.69E-05
	uberon 0003946	placenta labyrinth	0.00010402

Supplementary Table S23. The enriched Biological Process terms from the Gene Ontology for the zebrafish orthologs of the forelimb module-specific proteins.

Term identifier	Term name	P-value
GO:0006355	regulation of transcription, DNA-templated	2.74E-11
GO:0007275	multicellular organism development	2.01E-09
GO:0009953	dorsal/ventral pattern formation	1.36E-08
GO:0016055	Wnt signaling pathway	6.90E-08
GO:0006351	transcription, DNA-templated	2.18E-07
GO:0030509	BMP signaling pathway	5.94E-07
GO:0030182	neuron differentiation	7.04E-07
GO:0035118	embryonic pectoral fin morphoproteinsis	1.60E-05
GO:0045165	cell fate commitment	5.38E-05
GO:0030510	regulation of BMP signaling pathway	1.57E-04
GO:0048701	embryonic cranial skeleton morphoproteinsis	4.43E-04
GO:0002072	optic cup morphoproteinsis involved in camera-type eye development	4.70E-04
GO:0007178	transmembrane receptor protein serine/threonine kinase signaling pathway	0.00117724
GO:0040007	growth	0.00129193
GO:0060027	convergent extension involved in gastrulation	0.00135252
GO:0010862	positive regulation of pathway-restricted SMAD protein phosphorylation	0.0015685
GO:0042074	cell migration involved in gastrulation	0.00165586
GO:0048468	cell development	0.00172114
GO:0060395	SMAD protein signal transduction	0.00188379
GO:0030903	notochord development	0.00205679
GO:0043408	regulation of MAPK cascade	0.00205679
GO:0007179	transforming growth factor beta receptor signaling pathway	0.00414164
GO:0021592	fourth ventricle development	0.00421122
GO:0045743	positive regulation of fibroblast growth factor receptor signaling pathway	0.00536967
GO:0048703	embryonic viscerocranium morphoproteinsis	0.00585852
GO:0060070	canonical Wnt signaling pathway	0.0061092
GO:0001944	vasculature development	0.00649015
GO:0015012	heparan sulfate proteoglycan biosynthetic process	0.00665665
GO:0002062	chondrocyte differentiation	0.00665665
GO:0030097	hemopoiesis	0.00670985
GO:0008543	fibroblast growth factor receptor signaling pathway	0.0095973
GO:0007417	central nervous system development	0.01064297
GO:0061386	closure of optic fissure	0.01302316
GO:0006024	glycosaminoglycan biosynthetic process	0.01302316

GO:0071910	determination of liver left/right asymmetry	0.01490327
GO:0007064	mitotic sister chromatid cohesion	0.01689251
GO:0009948	anterior/posterior axis specification	0.01898788
GO:0051216	cartilage development	0.01918023
GO:0007155	cell adhesion	0.01955582
GO:0061371	determination of heart left/right asymmetry	0.02012978
GO:0045595	regulation of cell differentiation	0.02118641
GO:0030198	extracellular matrix organization	0.0234852
GO:0060026	convergent extension	0.02377358
GO:0038108	negative regulation of appetite by leptin-mediated signaling pathway	0.02505648
GO:2000366	positive regulation of STAT protein import into nucleus	0.02505648
GO:0060912	cardiac cell fate specification	0.02505648
GO:1900745	positive regulation of p38MAPK cascade	0.02505648
GO:2000583	regulation of platelet-derived growth factor receptor-alpha signaling pathway	0.02505648
GO:0002076	osteoblast development	0.02505648
GO:0070587	regulation of cell-cell adhesion involved in gastrulation	0.02505648
GO:1990051	activation of protein kinase C activity	0.02505648
GO:0043010	camera-type eye development	0.02506223
GO:0060041	retina development in camera-type eye	0.02513516
GO:0060828	regulation of canonical Wnt signaling pathway	0.02588139
GO:0001503	ossification	0.02588139
GO:0060037	pharyngeal system development	0.02837219
GO:0050767	regulation of neuroproteinsis	0.02837219
GO:0002138	retinoic acid biosynthetic process	0.03734953
GO:0032481	positive regulation of type I interferon production	0.03734953
GO:0010332	response to gamma radiation	0.03734953
GO:0032965	regulation of collagen biosynthetic process	0.03734953
GO:0006978	DNA damage response, signal transduction by p53 class mediator resulting in transcription of p21 class mediator	0.03734953
GO:0010470	regulation of gastrulation	0.03734953
GO:0071733	transcriptional activation by promoter-enhancer looping	0.03734953
GO:0030917	midbrain-hindbrain boundary development	0.04515412
GO:0001568	blood vessel development	0.04877064
GO:0048730	epidermis morphoproteinsis	0.0494884
GO:0032868	response to insulin	0.0494884

Supplementary Table S24. The enriched Uberon terms for the zebrafish orthologs of the forelimb module-specific proteins.

Uberon identifier	Term name	P-value
uberon 0008823	neural tube derived brain	2.29E-07
uberon 0000033	head	2.96E-07
uberon 0000468	multicellular organism	3.10E-07
uberon 0000019	camera-type eye	2.07E-05
uberon 0001555	digestive tract	9.84E-05
uberon 0001016	nervous system	0.00082865
uberon 0002107	liver	0.00093463
uberon 0001017	central nervous system	0.00141338
uberon 2000084	yolk	0.00145089
uberon 4000164	caudal fin	0.00325469
uberon 0000948	heart	0.00876155
uberon 0008895	splanchnocranium	0.01593721
uberon 0002280	otolith	0.01730733
uberon 0005281	ventricular system of central nervous system	0.02130098
uberon 2000033	intermediate cell mass of mesoderm	0.02183289
uberon 0001782	pigmented layer of retina	0.02256819
uberon 0001231	nephron tubule	0.02976965
uberon 0001155	colon	0.02976965
uberon 0001708	jaw skeleton	0.0342243
uberon 0005886	post-hyoid pharyngeal arch skeleton	0.03545473
uberon 0001647	facial nerve	0.03865184
uberon 0002407	pericardium	0.04825439

Supplementary Table S25. The enriched Biological Process terms from the Gene Ontology for the zebrafish orthologs of the hindlimb module-specific proteins.

Term identifier	Term name	P-value
GO:0006355	regulation of transcription, DNA-templated	1.34E-09
GO:0007275	multicellular organism development	5.81E-09
GO:0040007	growth	7.61E-07
GO:0010862	positive regulation of pathway-restricted SMAD protein phosphorylation	1.22E-06
GO:0060395	SMAD protein signal transduction	1.89E-06
GO:0043408	regulation of MAPK cascade	2.33E-06
GO:0048701	embryonic cranial skeleton morphoproteinsis	1.03E-05
GO:0048468	cell development	1.49E-05
GO:0051216	cartilage development	1.50E-05
GO:0030182	neuron differentiation	3.49E-05
GO:0016055	Wnt signaling pathway	5.61E-05
GO:0007417	central nervous system development	1.10E-04
GO:0060070	canonical Wnt signaling pathway	1.97E-04
GO:0006351	transcription, DNA-templated	2.70E-04
GO:0008543	fibroblast growth factor receptor signaling pathway	3.76E-04
GO:0001501	skeletal system development	5.14E-04
GO:0007155	cell adhesion	6.49E-04
GO:0048703	embryonic viscerocranium morphoproteinsis	9.92E-04
GO:0043401	steroid hormone mediated signaling pathway	0.00114721
GO:0030903	notochord development	0.00117063
GO:0005975	carbohydrate metabolic process	0.00129702
GO:0009612	response to mechanical stimulus	0.00135076
GO:0030510	regulation of BMP signaling pathway	0.00160837
GO:0030513	positive regulation of BMP signaling pathway	0.00204619
GO:0001503	ossification	0.00204619
GO:0045165	cell fate commitment	0.00207662
GO:0000165	MAPK cascade	0.00225021
GO:0002062	chondrocyte differentiation	0.00225021
GO:0007626	locomotory behavior	0.0023328
GO:0031101	fin regeneration	0.00239174
GO:0043010	camera-type eye development	0.0025896
GO:0007517	muscle organ development	0.00308006
GO:0009953	dorsal/ventral pattern formation	0.00369912
GO:0005978	glycogen biosynthetic process	0.0039571

GO:0030916	otic vesicle formation	0.0039571
GO:0035118	embryonic pectoral fin morphoproteinsis	0.00553237
GO:0007179	transforming growth factor beta receptor signaling pathway	0.00553237
GO:0030282	bone mineralization	0.00628086
GO:0060349	bone morphoproteinsis	0.00737634
GO:0030902	hindbrain development	0.00739625
GO:0070654	sensory epithelium regeneration	0.00769009
GO:0031099	regeneration	0.00927093
GO:0007519	skeletal muscle tissue development	0.0106517
GO:0043627	response to estrogen	0.01086062
GO:0061024	membrane organization	0.01086062
GO:0021587	cerebellum morphoproteinsis	0.01086062
GO:0043697	cell dedifferentiation	0.01086062
GO:0009948	anterior/posterior axis specification	0.01102739
GO:0042074	cell migration involved in gastrulation	0.01173397
GO:0045595	regulation of cell differentiation	0.01296272
GO:0048839	inner ear development	0.01440797
GO:0008045	motor neuron axon guidance	0.01440797
GO:0061300	cerebellum vasculature development	0.01492536
GO:0030198	extracellular matrix organization	0.01507944
GO:0060536	cartilage morphoproteinsis	0.01737942
GO:0060021	palate development	0.01953552
GO:0046716	muscle cell cellular homeostasis	0.01953552
GO:0060538	skeletal muscle organ development	0.01953552
GO:0048884	neuromast development	0.01986392
GO:0060037	pharyngeal system development	0.01986392
GO:0035567	non-canonical Wnt signaling pathway	0.02253359
GO:0042981	regulation of apoptotic process	0.02458938
GO:0072661	protein targeting to plasma membrane	0.02465756
GO:0006874	cellular calcium ion homeostasis	0.02703075
GO:0055113	epiboly involved in gastrulation with mouth forming second	0.02923793
GO:0016203	muscle attachment	0.0302594
GO:0030500	regulation of bone mineralization	0.0302594
GO:0048840	otolith development	0.03165263
GO:0007267	cell-cell signaling	0.03311103
GO:0001889	liver development	0.03497684
GO:0051482	positive regulation of cytosolic calcium ion concentration involved in phospholipase C-activating G-protein coupled signaling pathway	0.03505962
GO:0060027	convergent extension involved in gastrulation	0.03507928

GO:0001568	blood vessel development	0.03507928
GO:0055001	muscle cell development	0.03631032
GO:0030199	collagen fibril organization	0.03631032
GO:0060041	retina development in camera-type eye	0.03668877
GO:0006357	regulation of transcription from RNA polymerase II promoter	0.03680213
GO:0060059	embryonic retina morphoproteinsis in camera-type eye	0.03864788
GO:0045944	positive regulation of transcription from RNA polymerase II promoter	0.0397757
GO:0006508	proteolysis	0.04168701
GO:0007631	feeding behavior	0.04278097

Supplementary Table S26. The enriched Uberon terms for the zebrafish orthologs of the hindlimb module-specific proteins.

Uberon identifier	Term name	P-value
uberon 0000019	camera-type eye	7.22E-18
uberon 0000468	multicellular organism	5.69E-17
uberon 0000033	head	2.90E-15
uberon 0008823	neural tube derived brain	1.67E-12
uberon 0001016	nervous system	5.42E-10
uberon 0001017	central nervous system	1.81E-08
uberon 0002107	liver	4.19E-07
uberon 0000948	heart	6.07E-07
uberon 0001555	digestive tract	1.70E-06
uberon 0001032	sensory system	1.93E-06
uberon 0007812	post-anal tail	2.28E-06
uberon 0002407	pericardium	1.47E-05
uberon 0005886	post-hyoid pharyngeal arch skeleton	3.02E-05
uberon 2000084	yolk	3.05E-05
uberon 0005281	ventricular system of central nervous system	6.07E-05
uberon 0010314	structure with developmental contribution from neural crest	0.00074386
uberon 0001846	internal ear	0.0008888
uberon 0002329	somite	0.00089648
uberon 0001003	skin epidermis	0.00170376
uberon 0003102	surface structure	0.00190061
uberon 0002100	trunk	0.00252997
uberon 0001708	jaw skeleton	0.00287797
uberon 0004141	heart tube	0.00345967
uberon 0001782	pigmented layer of retina	0.00380629
uberon 0000017	exocrine pancreas	0.00384968
uberon 4000164	caudal fin	0.0046263
uberon 0001945	superior colliculus	0.00488419
uberon 0012438	blastema of regenerating fin/limb	0.00591299
uberon 0000965	lens of camera-type eye	0.00652003
uberon 0000966	retina	0.0085354
uberon 0002280	otolith	0.01008715
uberon 0008897	fin	0.01130278
uberon 0002028	hindbrain	0.01447067
uberon 0001890	forebrain	0.01678628

uberon 0014907	intersomitic vessel	0.01866955
uberon 0005884	hyoid arch skeleton	0.01978595
uberon 2000106	extension	0.02390583
uberon 0005310	pronephric nephron tubule	0.02958931
uberon 2001456	pectoral fin endoskeletal disc	0.0328624
uberon 0011611	ceratohyal bone	0.0328624
uberon 0003052	midbrain-hindbrain boundary	0.03515936
uberon 0002328	notochord	0.03668385
uberon 0002082	cardiac ventricle	0.03785239
uberon 0006283	future cardiac ventricle	0.04193983
uberon 0005087	tooth placode	0.04412975
uberon 0002422	fourth ventricle	0.04604822

Supplementary Table S27. Some of the enriched GO-BP and Uberon terms that are related to fin to limb transition for the zebrafish orthologs of the forelimb module-specific proteins. The terms are organized into specific anatomical regions.

Anatomical region	Term Identifier	Term name	P-value
Fin related	uberon 4000164	caudal fin	0.00325469
	GO:0035118	embryonic pectoral fin morphoproteinsis	1.60E-05
Other	uberon 0000033	head	2.96E-07
	uberon 0005886	post-hyoid pharyngeal arch skeleton	0.03545473
	uberon 0001708	jaw skeleton	0.0342243
	uberon 0008895	splanchnocranium	0.01593721
	uberon 0002280	otolith	0.01730733
	GO:0060037	pharyngeal system development	0.02837219

Supplementary Table S28. Some of the enriched GO-BP and Uberon terms that are related to fin to limb transition for the zebrafish orthologs of the hindlimb module-specific proteins. The terms are organized into specific anatomical regions.

Anatomical region	Term Identifier	Term name	P-value
Fin related	uberon 4000164	caudal fin	0.0046263
	uberon 0012438	blastema of regenerating fin/limb	0.00591299
	uberon 2001456	pectoral fin endoskeletal disc	0.0328624
	GO:0035118	embryonic pectoral fin morphoproteinsis	0.00553237
	GO:0031101	fin regeneration	0.00239174
Other	uberon 0000033	head	2.90E-15
	uberon 0005886	post-hyoid pharyngeal arch skeleton	3.02E-05
	uberon 0001708	jaw skeleton	0.00287797
	uberon 0005884	hyoid arch skeleton	0.01978595
	uberon 0011611	ceratohyal bone	0.0328624
	uberon 0002280	otolith	0.01008715
	GO:0060037	pharyngeal system development	0.02837219
	GO:0048701	embryonic cranial skeleton morphoproteinsis	1.03E-05
	GO:0048703	embryonic viscerocranium morphoproteinsis	9.92E-04
	GO:0060021	palate development	0.01953552
	GO:0060037	pharyngeal system development	0.01986392
	GO:0048840	otolith development	0.03165263
